# Erianin induces ferroptosis in GSCs via REST/LRSAM1 mediated SLC40A1 ubiquitination to overcome TMZ resistance

**DOI:** 10.1038/s41419-024-06902-4

**Published:** 2024-07-22

**Authors:** Maierdan Mansuer, Lin Zhou, Chengbin Wang, Liang Gao, Yang Jiang

**Affiliations:** Department of Neurosurgery, Shanghai Tenth People’s Hospital, Tongji University School of Medicine, Shanghai, 200072 China

**Keywords:** CNS cancer, Cancer stem cells

## Abstract

In recent studies, erianin, a natural product isolated from Dendrobium chrysotoxum Lindl, has exhibited notable anticancer properties. Ferroptosis, a novel form of programmed cell death, holds potential as a strategy to overcome Temozolomide (TMZ) resistance in glioma by inducing ferroptosis in TMZ-resistant glioma cells. Here, utilizing various phenotyping experiments, including cell counting kit-8 (CCK-8) assays, EdU assays, transwell assays, neurosphere formation assays and extreme limiting dilution (ELDA) assays, we demonstrated that erianin exerts its anticancer activity on both TMZ sensitive and TMZ-resistant glioma stem cells (GSCs). Furthermore, we made an exciting discovery that erianin enhances TMZ sensitivity in TMZ-resistant GSCs. Subsequently, we demonstrated that erianin induced ferroptosis in TMZ-resistant GSCs and enhances TMZ sensitivity through inducing ferroptosis, which was confirmed by intracellular measurements of ROS, GSH, and MDA, as well as through the use of BODIPY (581/591) C11 and transmission electron microscopy. Conversely, the ferroptosis inhibitor ferrostatin-1 (Fer-1) blocked the effects of erianin. The underlying mechanism of ferroptosis induced by erianin was further explored through co-immunoprecipitation (Co-IP) assays, ubiquitination assays, protein stability assessments, chromatin immunoprecipitation (ChIP) assays and luciferase reporter gene assays. We found that erianin specifically targets REST, inhibiting its transcriptional repression function without altering its expression levels. Consequently, this suppression of REST’s role leads to an upregulation of LRSAM1 expression. In turn, LRSAM1 ubiquitinates and degrades SLC40A1, a protein that inhibits ferroptosis by exporting ferrous ions. By downregulating SLC40A1, erianin ultimately induces ferroptosis in TMZ-resistant GSCs. Taken together, our research demonstrates that the natural product erianin inhibits the malignant phenotype of GSCs and increases the sensitivity of TMZ in TMZ-resistant GSCs by inducing ferroptosis. These findings suggest erianin as a prospective compound for the treatment of TMZ-resistant glioma.

## Introduction

Glioblastoma (GBM) is the most malignant primary tumor of the central nervous system, with a WHO grade of IV in all gliomas [1]. GBM exhibits infiltrative growth characteristics and often recurs shortly after surgery [2]. Even with aggressive surgical and postoperative systemic radiotherapy and chemotherapy, the median survival times of GBM patients is only 14.6 months [3]. Glioma stem cells (GSCs) represent a subpopulation of tumor cells within GBM that have stemness features, self-renewal, tumor-initiating ability, and clonal proliferative potential. GSCs contribute significantly to GBM heterogeneity, therapy resistance, recurrence, and metastasis [4]. Understanding the molecular mechanism of malignant phenotype of GSCs is crucial for exploring new therapeutic approaches to extend patient survival [5]. Temozolomide (TMZ) is a conventional chemotherapy agent for GBM, extend survival by approximately 2.5 months. However, TMZ resistance severely restricts the efficacy of TMZ chemotherapy [6]. Overcoming this resistance as well as finding suitable combination therapies to improve TMZ sensitivity is a pressing issue in clinical GBM treatment.

Dendrobium chrysotoxum Lindl (DCL) is a precious medicinal plant, distributed in southern China, southeast Asia, and India. Erianin, is a small molecular weight natural compound isolated from DCL with diverse pharmacological effects [7], including inducing cell apoptosis [8, 9], ferroptosis [10], cell cycle arrest [11] and acting as anti-angiogenesis [12] in various cancer types. However, its anticancer effects on glioma remain unknown, warranting a broader exploration of its pharmacological actions and mechanisms.

Ferroptosis is a novel form of cell death, distinct from other established forms such as necroptosis, apoptosis, and autophagy, characterized by high iron levels and lipid peroxidation-induced cell death [13]. Ferroptosis can bypass apoptosis resistance seen in traditional therapies, and recent studies have shown that cancer cells that are resistant to traditional therapies are more prone to occur ferroptosis [14–16]. Therefore, inducing ferroptosis is a potential strategy to overcome drug resistance for tumors. In addition, glioma cells exhibit increased iron uptake and high levels of free iron, indicating an inherent tendency towards ferroptosis [17, 18]. However, the role of ferroptosis in TMZ-resistant glioma remains to be explored.

In this study, we investigated the impact of erianin on the viability of two glioblastoma cell lines and two GSCs extracted from our GBM patients. Notably, we demonstrated for the first time that erianin inhibited GSCs viability, inhibited cell proliferation by G2/M phase arrest, and simultaneously suppressed invasion, migration, and neurosphere-forming abilities of GSCs. Meantime, we found that erianin also exhibited significant inhibitory activity against TMZ-resistant GSCs and increases TMZ sensitivity when used in combination with TMZ via inducing ferroptosis in TMZ-resistant GSCs. Subsequently, we identified REST/LRSAM1 mediated SLC40A1 ubiquitination as the key mediator of erianin-induced ferroptosis. In conclusion, our results suggest that the natural compound erianin exerts its anti-GSCs and anti TMZ-resistant effects through inducing SLC40A1 ubiquitination-dependent ferroptosis.

## Methods

### Glioma tissue specimen collection and ethical approval

Total of 70 glioma tissue specimens were obtained from patients diagnosed with glioma and undergoing surgery at the Neurosurgery Department of the Shang-Hai Tenth People’s Hospital from January 2015 to December 2019. All pathological specimens underwent histopathological examination. According to the World Health Organization (WHO) classification guidelines, 18 samples were classified as grade II gliomas, 24 as grade III gliomas, and 18 as grade IV gliomas. Additionally, ten adjacent normal brain tissues (NBT) from glioblastoma (GBM) patients were collected as normal controls. All participants provided written informed consent, and the study received approval from the Ethics Committee of the Shanghai Tenth People’s Hospital.

### Cell lines and reagents

Human glioblastoma U87 and U118 cell lines were purchased from the Chinese Academy of Sciences Cell Bank. The cells were maintained in DMEM medium with 10% fetal bovine serum (FBS) at 37 °C in a humidified atmosphere with 95% air and 5% CO_2_. Purified erianin (>98%) (#95041) was obtained from Shanghai YuanYe Biotechnology (China), and a 25 mM stock solution was prepared using DMSO.

### GSC cell culture

GSCs were isolated and validated from WHO grade IV specimens (GSCm01, GSCm03) from two patients. Table S1 provides detailed clinical information on these samples. In brief, fresh GBM tissue was dissociated into single cells using type ||collagenase. The dissociated cells were maintained in serum-free DMEM/F12 supplemented with 2% B27, 20 ng/mL rh-bFGF, and rh-EGF (Gibco, USA) for two weeks until tumor sphere formed. The cells were tested for stem cell markers and multi-lineage differentiation to verify GSC characteristics. All GSCs were underwent mycoplasma detection and short tandem repeat (STR) DNA analysis and cultured for fewer than 20 passages.

### U87R and GSCm01R cell lines

U87 cells and GSCm01 cells were cultured in 96-well plates at a density of 6000 cells/well. The half-maximal inhibitory concentration (IC50) of TMZ for U87 and GSCm01 was calculated using the CCK-8 assay. Subsequently, U87 and GSCm01 were cultured in media containing TMZ (at a concentration of IC50/10) for 14 days, maintaining a stable growth. When the cells grow stably, double the dose of TMZ is given, and each dose remains unchanged for 14 days. After four months, the TMZ-resistant GBM cells were successfully induced and named U87R and GSCm01R cells.

### Lentiviral vector construction and transfection

Gene-Chem (China) lentiviral vectors were used to construct permanent overexpression and corresponding negative controls for REST, LRSAM1, and SLC40A1. Lentiviral vectors for RNA interference (RNAi) targeting REST and LRSAM1 were constructed using Gene-Chem’s RNAi-mediated lentiviral vectors. The effectiveness of lentiviral transfection was verified by qPCR or western blotting assays. The RNAi sequences used in this study are listed in Table S2.

### Quantitative real-time PCR(RT-qPCR) assay

Total RNA was extracted using TRIzol (Invitrogen, USA), and cDNA libraries were constructed using the Prime-Script RT Master Mix Kit (TaKaRa, Japan). RT-qPCR was performed using the SYBR Green Master Mix Kit (TaKaRa) on the Mx-3000P Quantitative PCR System (Applied Biosystems, USA). The 2^−ΔΔCt^ method was used to calculate and process the obtained data, with GAPDH expression serving as the endogenous control to manifest the relative gene expression levels. The primers used in this study were purchased from Sangon Biotech (China) and the sequences are listed in Table S3.

### Western blotting assay

Cells from diverse groups were lysed in RIPA buffer containing 1% protease and phosphatase inhibitors (Beyotime Biotechnology, China). Protein quantification and denaturation were performed, and proteins were separated by SDS-PAGE gel electrophoresis and transferred onto PVDF membranes. The membranes were then incubated overnight at 4 °C with primary antibodies targeting the specific proteins of interest. After washing off the primary antibodies, secondary antibodies were added, and the incubation continued at room temperature for 1 h. Protein bands were visualized using the chemiluminescence ECL kit (YEASEN, China) and a chemiluminescence imaging system (Tanon, China). The gray values of protein bands were quantified using Image J software (National Institutes of Health, USA). Antibodies used in this study are listed in the Table S4.

### Measurement of the cell inhibition rate, apoptosis, and cell cycle

The cell inhibition rate was assessed using the Cell Counting Kit-8 (CCK-8) assay (Beyotime Biotechnology). Cell apoptosis was detected using the Annexin V-FITC/PI Apoptosis Kit (Multi Sciences, China) by flow cytometry, and the cell cycle was detected using the Cell Cycle Staining Kit (Multi Sciences) with a flow cytometry analyzer (BD, USA).

### Neurosphere formation assay (NSFA)

Neurosphere formation was assessed as previously described [19]. In brief, GSCs were cultured at a density of 10^3^ cells/well in a 6-well plate and cultured for 7 days. After neurosphere formed, the images of neurospheres were obtained using an optical microscope (Olympus, Japan), and the relative diameters were recorded for subsequent analysis calculations.

### Extreme limiting dilution assay (ELDA)

To assess GSC neurosphere-forming ability, an ELDA was conducted as described earlier [19]. GSCs were cultured at densities of 1, 10, 20, 30, 40, and 50 cells/well in a 96-well plate, with each density repeated 10 times. After 7 days, the number of wells with neurosphere formation (NSF) was observed using an inverted optical microscope (Olympus). The neurosphere synthesis capacity was analyzed via using extreme limiting dilution analysis (ELDA, http://bioinf.wehi.edu.au/software/elda).

### Measurement of lipid-reactive oxygen species (ROS)

The intracellular ROS levels were detected using the Reactive Oxygen Species Assay Kit (Beyotime Biotechnology). U87R and GSCm01R cells were collected, suspended in the diluted probe solution, and incubated at 37 °C for 20 min. After washing, cells were subjected to flow cytometry (BD) to detect ROS levels in the FITC spectrum.

### Glutathione (GSH) and malondialdehyde (MDA) assay

The GSH content in U87R and GSCm01R was measured using a reduced glutathione (GSH) assay kit (Colorimetric, Sigma-Aldrich, China). According to the manufacturer’s instructions, cells were harvested, and a reaction was initiated with the assay reagents. Finally, the GSH content was reflected by measuring the absorbance of the reaction product at 450 nm using a UV spectrophotometer (Thermo Fisher Scientific, USA). Similarly, the MDA content in U87R and GSCm01R cells was detected using an MDA assay kit (Colorimetric/Fluorometric, Abcam, USA). In the lipid peroxidation assay, MDA in cells reacted with thiobarbituric acid (TBA) to form the MDA-TBA adduct, and quantification was performed at 532 nm.

### C11-BODIPY (581/591) assay

The ferroptosis levels were evaluated using the BODIPY 581/591 C11 kit (Thermo Fisher Scientific). U87R and GSCm01R cells were cultured at a density of 5 × 10^4^ cells/well in a 6-well plate and cultured for 24 h. Subsequently, cells were stained with 2 μM C11-BODIPY (581/591) probe according to the manufacturer’s instructions. Visualization was done using a confocal microscope (LSM 880, Carl Zeiss AG, Germany), and analysis was performed using Image J software (NIH, USA). Oxidized BODIPY (O-BODIPY) was observed at an excitation/emission wavelength of 488/510 nm (traditional FITC filter set), while reduced BODIPY (R-BODIPY) was observed at 581/591 nm (Texas Red filter set).

### Small-molecule pull-down

Biotin-labeled erianin was acquired from PSI Biotechnology [20]. U87R and GSCm01R cells were harvested and lysed in RIPA buffer supplemented with protease and phosphatase inhibitors with brief sonication. After centrifugation at 12,000 × *g* for 30 min, the supernatant was collected. Then, the samples were incubated with streptavidin beads, biotin-labeled erianin, and with and without the presence of unlabeled erianin (tenfold of biotin-labeled erianin) in RIPA buffer overnight at 4 °C, respectively. After incubation, the beads were washed three times with RIPA buffer, and the bead-bound proteins were eluted, separated by SDS-PAGE, and visualized by western blotting.

### Co-immunoprecipitation (CO-IP)

The Co-ip assays were analyzed using the Pierce Classic Magnetic IP/Co-IP Kit (Thermo Fisher Scientific). U87R and GSCm01R cells were lysed in IP lysis buffer containing protease and phosphatase inhibitors. According to the manufacturer’s protocol, cell lysates were incubated with antibody-coupled magnetic beads overnight at 4 °C. Immunoprecipitated proteins and total cell lysates were collected for western blotting to analyze protein interactions.

### Protein stability assessment

U87R and GSCm01R cells were treated with 50 μM proteasome inhibitor MG132 (Sigma-Aldrich) for 6 h. After protein extraction, western blotting assays was performed to detect the expression of SLC40A1. Additionally, cells were treated with 50 μg/ml cycloheximide (Sigma-Aldrich), an inhibitor for neo-proteins synthesis, and lysed to harvest proteins at 2, 4, 6, 8, and 12 h for protein half-life assay through western blotting detection.

### Ubiquitination assay

Initially, U87R and GSCm01R cells were transfected with Flag-SLC40A1 using Lipofectamine 3000 (Invitrogen). Subsequently, cells were treated with 50 μM MG132 for 6 h, and total cell proteins were extracted using the Total Cell Protein Extraction Kit (KeyGen Biotechnology, China). The ubiquitination of SLC40A1 was detected by immunoprecipitation using an anti-Flag antibody (Abcam), followed by western blotting with an anti-Ubiquitin antibody (Abcam).

### Luciferase reporter assay

The purpose of the luciferase reporter gene assay is to analyze the transcriptional regulation of target genes by transcription factors. The complete nucleotide sequence of the LRSAM1 promoter was cloned into the pGL3 vector as a wild-type (LRSAM1-wt), and a mutant clone with REST binding site mutations in the promoter region (LRSAM1-mt) was generated. U87R and GSCm01R cells were cultured at a density of 5 × 10^3^ cells/well in a 96-well plate and transfected with pGL3 control plasmid/LRSAM1-wt plasmid/LRSAM1-mt plasmid. After 24 h, cells were lysed, and luciferase activity was detected and analyzed using the luciferase reporter gene assay kit (Beyotime Biotechnology) according to the operation manual.

### Chromatin immunoprecipitation (ChIP) assay

ChIP assays were conducted using the ChIP assay kit (Beyotime Biotechnology) according to the manufacturer’s instructions. In brief, chromatin complexes were immunoprecipitated using an anti-REST antibody. DNA was then extracted and purified from the complexes, and analyzed by qPCR. The primer sequences for ChIP qPCR are listed in Table S5.

### Measurement of intracellular Fe^2+^

The concentration of intracellular labile ferrous (Fe^2+^) ions in U87R and GSCm01R cells was detected using the FerroOrange kit (Dojindo, Japan) according to the manufacturer’s instructions. FerroOrange is a live cell imaging probe specifically designed to detect Fe^2+^ ions. Briefly, treated U87R and GSCm01R cells were co-incubated with 1 μM FerroOrange probe in a 96-well plate at 37 °C, 5% CO_2_ for 30 min, and the fluorescence intensity of each sample was measured using a multifunctional microplate reader (Ex: 543 nm/Em: 580 nm).

### In vivo tumor model

6-week-old female BALB/c nude mice were purchased from Shanghai Jihui Experimental Animal Care Co., Ltd. (China). All mice were housed under specific pathogen-free conditions at the Experimental Animal Center of the Tenth People’s Hospital of Shanghai [19]. The experiments were approved by the Animal Protection Committee of the Tenth People’s Hospital of Shanghai. Briefly, each group included five mice, and after anesthesia, 5 × 10^4^ GSCs were injected into the mice brain using a stereotactic apparatus by puncturing at the 2.5 mm lateral and 0.5 mm posterior to intersection of the coronal and sagittal sutures on the skull. Subsequently, the survival time of each mouse was calculated, and tumor size was recorded using an in vivo bioluminescence imaging system.

### Immunohistochemistry (IHC)

IHC was performed using an immunohistochemical labeling kit (Immunoway Biotechnology, USA). In summary, paraffin-embedded tumor sections were labeled overnight at 4 °C with primary antibodies against REST (1:100; Abcam), Ki-67 (1:100, Abcam), LRSAM1 (1:100, Abcam), SLC40A1(1:100, Abcam). The sections were then incubated with secondary antibodies from the IHC kit at 37 °C for 30 min, followed by imaging under an optical microscope (Olympus).

### Bioinformatics analysis

The basic information of erianin comes from pubCHEM (http://pubchem.ncbi.nlm.nih.gov/), and the binding between erianin and proteins is predicted using network pharmacology databases such as BindingDB, CHEMBL, HERB, PubChem, STITCH, and target prediction tools including BATMAN-TCM, PharmMapper, Chemmapper, SEA, SuperPred, SwissTargetPrediction, and TargetHunter. Ferroptosis-related genes were obtained through gene set enrichment analysis (GSEA) available at http://www.broadinstitute.org/gsea/index.jsp. Expression data for REST, LRSAM1, SLC40A1, and clinical information of GBM patients were sourced from the cancer genome atlas (TCGA) in the HG-U133A platform (http://cancergenome.nih.gov) and the Chinese Glioma Genome Atlas (CGGA) 325 RNA-seq platform (http://www.cgga.org.cn).

### Statistical analysis

Statistical analyses were performed by using R version 4.2.1 and GraphPad Prism version 9.0 software. All experiments were repeated at least three times, and results were expressed as mean ± standard deviation. Statistical significance among different groups was assessed using two-tailed Student’s *t*-test, chi-square test, or one-way analysis of variance (ANOVA). Kaplan–Meier analysis was employed to plot survival curves, and log-rank tests were conducted for evaluation. *P* values < 0.05 were accepted as statistically significant (*p < 0.05; **p < 0.01; ***p < 0.001).

## Result

### Erianin inhibits malignant progression of glioblastoma

**Fig. 1:**
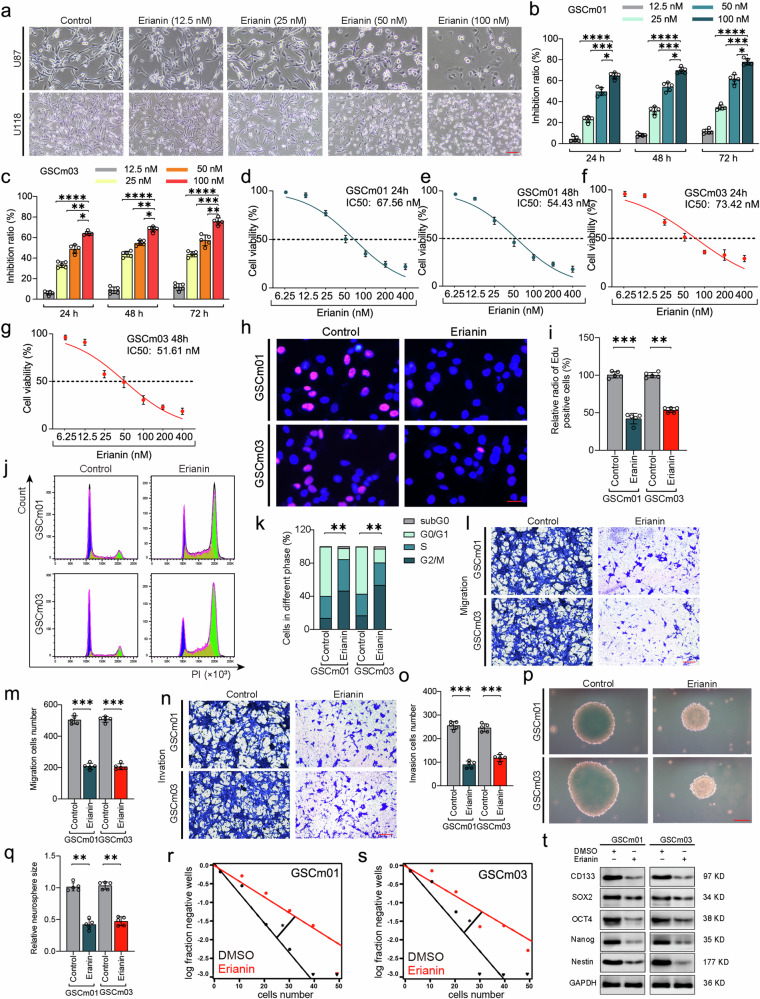
Erianin inhibits the malignant progression of GBM. **a** Morphological changes in representative U87 and U118 cells after treatment with different concentrations of erianin. Scale bar = 50 μm. **b**, **c** Cell viability of GSCm01 and GSCm03 after treatment with erianin at different times and concentrations, measured using CCK-8. **d**–**g** IC50 of GSCm01 and GSCm03 at 24 and 48 h after treatment with erianin, measured using CCK-8. **h**, **i** Representative EdU assay showing proliferation and quantitative analysis of GSCm01 and GSCm03 after erianin treatment. Scale bar = 50 μm. **j**, **k** Representative results of cell cycle assay and quantitative analysis of GSCm01 and GSCm03 after erianin treatment. **l**, **m** Migration assays of GSCm01 and GSCm03 using a Transwell 24-well plate without extracellular matrix gel, and quantitative analysis after erianin treatment. Scale bar = 50 μm. **n**, **o** Invasion assays of GSCm01 and GSCm03 using a Transwell 24-well plate with extracellular matrix gel, and quantitative analysis after erianin treatment. Scale bar = 50 μm. **p**, **q** NSFA revealing the size of neurospheres and quantitative analysis of GSCm01 and GSCm03 after erianin treatment. Scale Bar = 50 μm. **r**, **s** ELDA showing the neurosphere-forming ability of GSCm01 and GSCm03 after erianin treatment. **t** Western blotting of the stemness marker in GSCm01 and GSCm03 after erianin treatment. All data are shown as the mean ± SD (five independent experiments). *p < 0.05; **p < 0.01; ***p < 0.001; ns no significance.

To investigate the cytotoxic and inhibitory effects of erianin on GBM cells, different concentrations of erianin were treated to U87, U118, GSCm01, and GSCm03 cells for different times. Optical microscope observation shows that the increase in erianin concentration leads to the destruction of U87 and U118 cell morphology and a significant reduction in cells number (Fig. 1a). Subsequent CCK-8 assays showed that erianin significantly inhibited the viability of GSCm01 and GSCm03 cells, with inhibitory capacity dependent on both time and concentration (Fig. 1b, c). Repeating CCK-8 assays on U87 and U118 cells produced similar results (Supplementary Fig. 1a, b). The IC50 obtained by CCK-8 assays further indicated that erianin already exhibited significant GBM cell inhibitory ability after 24 h of treatment (Fig. 1d–g; Supplementary Fig. 1c–f). Consequently, the IC50 concentration of erianin at 24 h was selected as treatment concentration for subsequent EdU, transwell, and neurosphere formation assays. Next, to determine the anti-proliferation effect of erianin, EdU assays was performed. The results showed a significant decrease of EdU positive cells in GSCm01 and GSCm03 after erianin treatment, indicating that erianin treatment inhibited the proliferation of GSCs (Fig. 1h, i) To further confirm whether erianin inhibits cell proliferation by inducing cell cycle arrest, flow cytometry assays were performed. The results showed that erianin promoted G2/M phase arrest, accompanied by a decrease in the number of cells in the G0/G1 and S phases (Fig. 1j, k). The above results indicated that erianin treatment can effectively inhibit the viability and proliferation of GSCs.

To investigate whether erianin affects migration and invasion abilities of GSCs, transwell assays were performed. As shown in (Fig. 1l–o), the results showed that erianin treatment significantly reduced the number of invading and migrating cells in GSCm01 and GSCm03, with similar results obtained in U87 and U118 cells in the repeated experiments (Supplementary Fig. 1g–n). Subsequently, NSFA and ELDA were performed to investigate the effect of erianin on the neurosphere-forming ability of GSCs. The results showed a decrease in the relative size and formation ability of neurospheres after treatment with erianin in GSCm01 and GSCm03 (Fig. 1p–s). We next detected the expression of stem cell markers such as NESTIN, NANOG, OCT4, CD133, and SOX2 after erianin treatment in GSCm01 and GSCm03. We found that erianin suppressed the stemness of GSCs (Fig. 1t). Taken together, the experimental results showed that erianin treatment significantly inhibits the cell viability and malignant phenotype of GSCs, as well as suppressing the stemness capability of GSCs.

### Erianin promotes TMZ sensitivity in TMZ-resistant GSCs

**Fig. 2:**
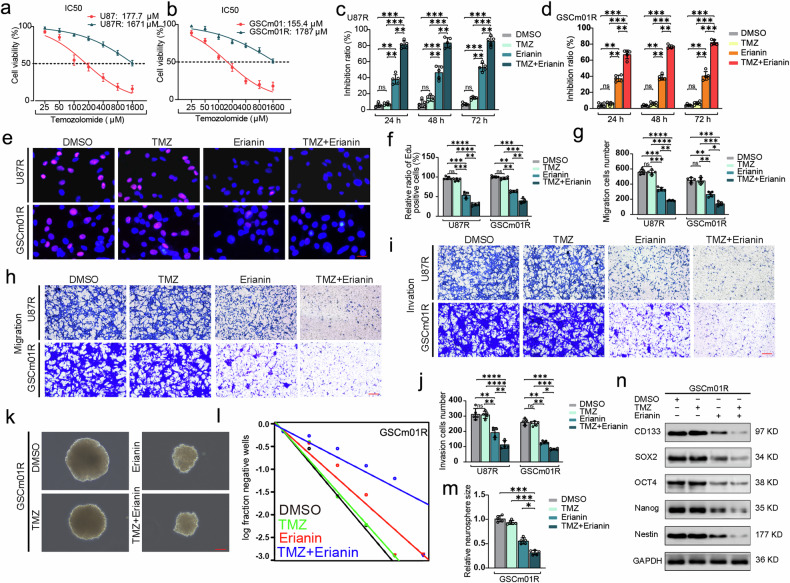
Erianin promotes TMZ sensitivity in TMZ-resistant GSCs. **a**, **b** IC50 of U87, GSCm01, and our established TMZ-resistant U87R, GSCm01R at 24 h after with TMZ treatment, measured using CCK-8. **c**, **d** Cell viability measured using CCK-8 after 24, 48, and 72 h of treatment with TMZ, erianin, and their combination in U87R and GSCm01R. **e**, **f** Representative EdU assay showing proliferation and quantitative analysis of U87R and GSCm01R after different treatment groups. Scale bar = 50 μm. **g**, **h** Migration assays of U87 and U118 using a Transwell 24-well plate without extracellular matrix gel, and quantitative analysis after different treatment groups. Scale bar = 50 μm. **i**, **j** Invasion assays of U87 and U118 using a Transwell 24-well plate with extracellular matrix gel, and quantitative analysis after different treatment groups. Scale bar = 50 μm. **k**, **m** NSFA showing the size of neurospheres and quantitative analysis of GSCm01R after different treatment groups. Scale bar = 50 μm. **l** ELDA showing the neurosphere-forming ability of GSCm01R after different treatment groups. **n** Western blotting of stemness marker in GSCm01R after different treatment groups. All data are shown as the mean ± SD (five independent experiments). *p < 0.05; **p < 0.01; ***p < 0.001; ns no significance.

In order to study the role of erianin in TMZ-resistant GSCs, we first established TMZ-resistant cell lines named U87R and GSCm01R based on U87 and GSCm01 cells. The IC50 of TMZ increased by 9.4 times and 11.5 times in U87R and GSCm01R, respectively, compared to U87 and GSCm01(Fig. 2a, b). Indicating successful establishment of TMZ resistance. Subsequent CCK-8 assays showed that treatment of erianin alone inhibited the viability of U87R and GSCm01R cells, while TMZ alone showed almost no inhibitory effect on cell viability, result also showed that there is a significant inhibitory effect when erianin and TMZ are used in combination, indicating that erianin may promotes TMZ sensitivity in TMZ-resistant GSCs (Fig. 2c, d). EdU assays further revealed a significant reduction of EdU-positive cells, when erianin was combined with TMZ in the treatment of U87R and GSCm01R cells (Fig. 2e, f). Transwell assays confirmed that erianin combined with TMZ significantly reduced the migration and invasion abilities of U87R and GSCm01R cells (Fig. 2g–j). NSFA and ELDA demonstrated a decrease in the relative size and neurosphere-forming ability after erianin combined with TMZ treatment in GSCm01R (Fig. 2k–m). Western blotting results showed a decrease in GSC stemness markers after erianin combined with TMZ treatment in GSCm01R (Fig. 2n). In conclusion, erianin treatment can also effectively inhibits the malignant phenotype of TMZ-resistant GSCs, and with a more significant inhibition effect when combined with TMZ, suggesting that erianin may promotes TMZ sensitivity in TMZ-resistant GSCs.

### Erianin induces ferroptosis in TMZ-resistant GSCs

**Fig. 3:**
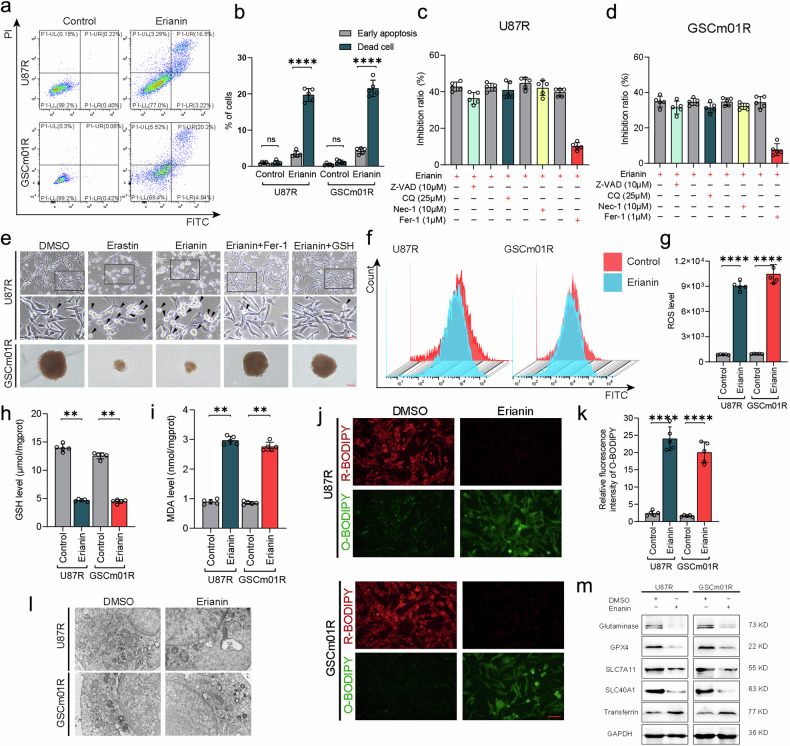
Erianin induces ferroptosis in TMZ-resistant GSCs. **a**, **b** Analysis of cell death and apoptosis in U87R and GSCm01R after erianin treatment, using annexin V FITC/PI staining. **c**, **d** Inhibitory measured using CCK-8 in U87R and GSCm01R cells 24 h after treatment with erianin and with or without Z-VAD-FMK, CQ, Nec-1, Fer-1. **e** Representative images of U87R cell morphological changes and GSCm01R neurosphere formation size with erastin treatment, erianin treatment, and subsequent Fer-1 and GSH treatment. Scale bar = 100 μm. **f**, **g** Measurement of ROS levels in U87R and GSCm01R after erianin treatment, using flow cytometry. **h** Measurement of intracellular GSH levels in U87R and GSCm01R treated with erianin. **i** Measurement of intracellular MDA levels in U87R and GSCm01R treated with Erianin. **j**, **k** ferroptosis detection in U87R and GSCm01R treated with Erianin, using BODIPY (581/591) C11 probe, and quantification of relative fluorescence intensity by Image J (**k**). Scale bar = 100 μm. **l** TEM used to observe ferroptosis in U87R and GSCm01R treated with Erianin (original magnification: ×100). **m** Expression of key ferroptosis regulators detected by western blotting in U87R and GSCm01R treated with Erianin. All data are shown as the mean ± SD (five independent experiments). *p < 0.05; **p < 0.01; ***p < 0.001; ns no significance.

As the diversity of cell death types increases, tumor cell death is no longer limited to traditional necrosis and apoptosis. New forms of cell death, such as ferroptosis, cuproptosis, pyroptosis, and disulfidptosis, have been discovered. We further investigated the mechanism of erianin-induced cell death and the inhibition of its malignant phenotype in TMZ-resistant GSCs. An annexin V-FITC dual staining assay was performed by flow cytometry, as a result, a significantly increased number of dead cells and minimal number of apoptosis was observed in U87R and GSCm01R after treatment with erianin (Fig. 3a, b), it suggested that nonapoptotic cell death occurred. Consequently, to identify the type of cell death induced by erianin, we treated cells with inhibitors of common cell death types after erianin treatment, including necrostatin-1 (necroptosis inhibitor, 10 μM), Z-VAD-FMK (a pancaspase inhibitor, 10 μM), CQ (effective autophagy inhibitor, 25 μM), and ferrostatin-1 (Fer-1, ferroptosis inhibitor, 10 μM). The results showed that necrostatin-1, Z-VAD-FMK, and CQ did not prevent erianin-induced cell death in U87R and GSCm01R, while ferrostatin-1 treatment significantly reversed the inhibitory effect of erianin in U87R and GSCm01R (Fig. 3c, d). Cell morphology observation also indicated that the ferroptosis inhibitor ferrostatin-1 and GSH (glutathione, 1 mM, a well-known ROS scavenger) could reverse erianin-induced cell death (Fig. 3e). This suggests that erianin-induced cell death in TMZ-resistant GSCs may be ferroptosis.

It is well known that lipid peroxidation, ROS accumulation, and GSH depletion are crucial events in ferroptosis. To further confirm our hypothesis, we conducted intracellular ROS detection in U87R and GSCm01R. The results showed a significant increase in ROS levels in U87R and GSCm01R cells after erianin treatment (Fig. 3f, g). We also measured the lipid peroxidation markers GSH and MDA (Fig. 3h, i), and the results indicated a decrease in GSH levels and an increase in MDA levels after erianin treatment in U87R and GSCm01R, confirming changes consistent with ferroptosis. Moreover, we used the BODIPY (581/591) C11 probe to detect the accumulation of lipid ROS, and the results showed a significant increase in green fluorescence, indicating an upregulation of lipid peroxidation after erianin treatment in U87R and GSCm01R cells (Fig. 3j, k). Mitochondrial damage leads to the accumulation of lipid peroxidation. Transmission electron microscopy (TEM) showed that erianin caused cell mitochondrial shrinkage, outer mitochondrial membrane (OMM) rupture, reduced cristae, and potential mitochondrial collapse, characteristic changes of ferroptosis (Fig. 3l). Finally, after erianin treatment, we used western blotting to detect changes in some important ferroptosis related proteins in U87R and GSCm01R. The results showed an increase in the expression of transferrin after erianin treatment, which is a positive regulatory protein of ferroptosis. Conversely, the expression of ferroptosis-negative regulatory proteins, such as GPX4, SLC40A1, SLC7A11, and glutaminase significantly decreased after erianin treatment (Fig. 3m). In summary, these findings strongly suggest that erianin induces ferroptosis in TMZ-resistant GSCs.

### Erianin promotes TMZ sensitivity in TMZ-resistant GSCs through ferroptosis

**Fig. 4:**
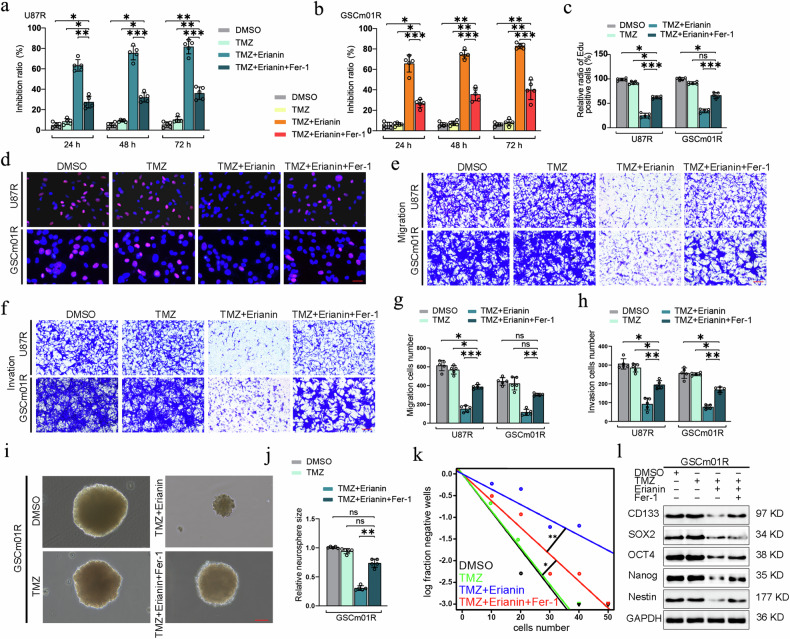
Erianin promotes TMZ sensitivity in TMZ-resistant GSCs through ferroptosis. **a**, **b** Cell viability of U87R and GSCm01R measured using CCK-8 after treatment with TMZ and erianin combined with Fer-1 at different times. **c**, **d** Representative EdU assay showing proliferation and quantitative analysis of U87R and GSCm01R after treatment with TMZ and erianin followed by Fer-1. Scale bar = 50 μm. **e**, **g** Migration assays of U87R and GSCm01R using a Transwell 24-well plate without extracellular matrix gel and quantitative analysis after treatment with TMZ and erianin combined with Fer-1. Scale bar = 50 μm. **f**, **h** Migration assays of U87R and GSCm01R using a Transwell 24-well plate with extracellular matrix gel and quantitative analysis after treatment with TMZ and erianin combined with Fer-1. Scale bar = 50 μm. **i**, **j** NSFA showing the size of neurospheres and quantitative analysis of GSCm01R after treatment with TMZ and erianin combined with Fer-1. Scale bar = 50 μm. **k** ELDA showing the neurosphere-forming ability of GSCm01R after treatment with TMZ and erianin combined with Fer-1. **l** Western blotting of stemness marker in GSCm01R after treatment with TMZ and erianin combined with Fer-1. All data are shown as the mean ± SD (five independent experiments). *p < 0.05; **p < 0.01; ***p < 0.001; ns no significance.

To further investigate whether the combination of erianin and TMZ promotes the TMZ sensitivity in U87R and GSCm01R by inducing ferroptosis, we conducted cell malignant phenotype assays after administering the ferroptosis inhibitor Fer-1 on the basis of erianin and TMZ co-treatment. CCK-8 assays showed that, after co-treatment with erianin and TMZ, the viability of U87R and GSCm01R cells was significantly inhibited. However, after adding Fer-1 treatment, cell viability was significantly recovered (Fig. 4a, b). EdU assay showed a significant reduction in EdU-positive cells after erianin and TMZ co-treatment, but this effect was reversed by Fer-1 treatment (Fig. 4c, d).

Subsequently, transwell assays further confirmed that erianin combined with TMZ significantly reduced the number of migrated and invaded U87R and GSCm01R cells. However, after Fer-1 treatment, the number of migrated and invaded cells increased (Fig. 4.e–h). Additionally, NSFA and ELDA assays explored whether Fer-1 could reverse the inhibitory effects of erianin and TMZ on neurosphere-forming ability of GSCm01R. Results showed that erianin combined with TMZ reduced the relative size and number of neurospheres in GSCm01R, and additional Fer-1 treatment increased their size and number (Fig. 4i–k). Finally, western blotting detected changes in stemness markers of GSCm01R, erianin combined with TMZ reduced the expression of NESTIN, NANOG, OCT4, CD133, and SOX2, while additional Fer-1 treatment increased their expression (Fig. 4l). Our results suggest that the inhibitory effects of erianin combined with TMZ on GSCs can be reversed by Fer-1 treatment, indicating that combination of erianin and TMZ promotes TMZ sensitivity through ferroptosis in U87R and GSCm01R.

### REST is the target of erianin in regulating gliomas, overexpressed in glioma and correlating with poor patient prognosis

**Fig. 5:**
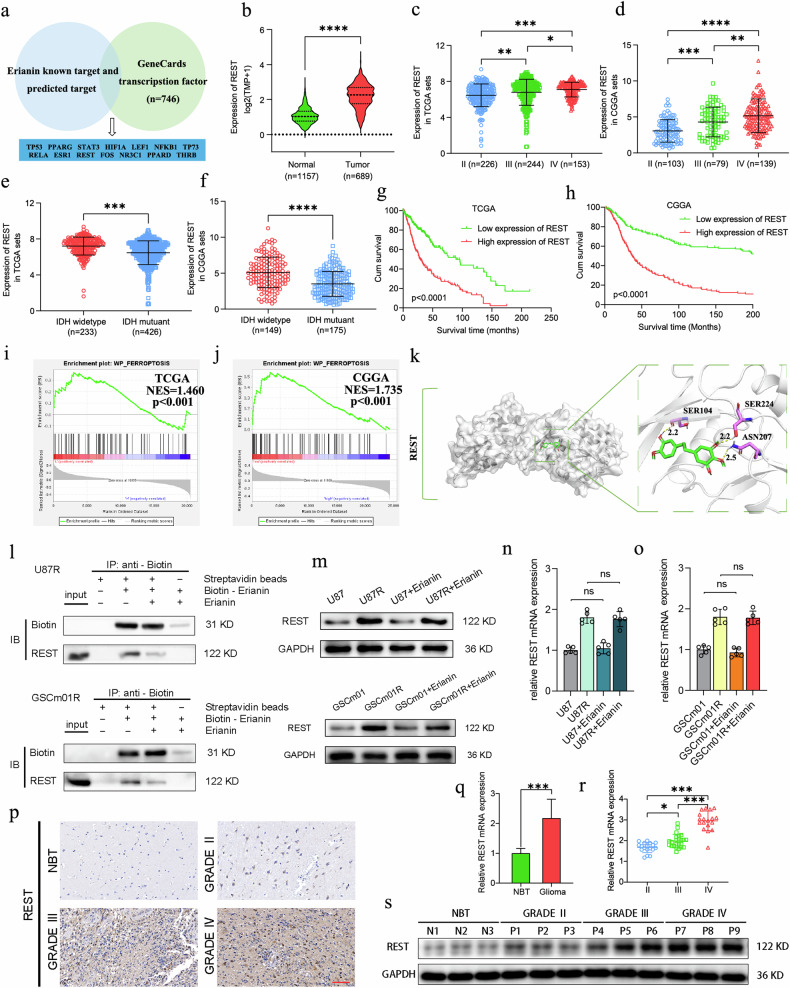
REST is the target of erianin, overexpressed in glioma and correlating with poor patient prognosis. **a** Venn chart for downstream target screening of erianin. **b** REST expression correlated with NBT and glioma based on GTEX and TCGA datasets. **c**, **d** REST expression correlated with glioma WHO grades based on TCGA and CGGA datasets. **e**, **f** REST expression correlated with glioma IDH mutation based on TCGA and CGGA datasets. **g**, **h** Kaplan–Meier survival analysis for glioma patients with high and low REST expression based on TCGA and CGGA datasets. **i**, **j** GSEA analysis of the correlation between REST expression and ferroptosis pathways in TCGA and CGGA databases. **k** Molecular Docking showing REST as the most likely predicted target of erianin. **l** U87R and GSCm01R cell lysates were incubated with biotin-erianin in the absence or presence of a 10 fold excess of unlabeled erianin, followed by pull-down with streptavidin-agarose. The precipitates were resolved by SDS-PAGE, and the gel was detected by western blotting. Western blot and qPCR assays showed REST protein expression (**m**) and mRNA expression (**n**, **o**) before and after erianin treatment. **q**, **r** qPCR assays showing mRNA expression of REST in glioma tissues (*n* = 70) and normal brain tissues (*n* = 10) (**l**), and in different WHO-grade glioma tissues (**m**). **p**, **s** Western blotting (**n**) and immunohistochemistry assays (**o**) showing REST expression in different WHO-grade glioma tissues. Scale bar = 50 μm. All data are shown as the mean ± SD (three independent experiments). *p < 0.05; **p < 0.01; ***p < 0.001.

To explore the possible mechanism of erianin-induced ferroptosis in TMZ-resistant GSCs, we conducted a multi-dimensional network pharmacology analysis. By referencing pharmacological databases and utilizing drug target prediction tools based on the structure of erianin, we identified potential binding targets for erianin (Table S6). Considering the crucial regulatory roles of transcription factors in various cancer-related pathways, especially in the occurrence and development of cancer, we intersected the potential binding targets of erianin with the transcription factor gene set from GeneCards (Table S7), resulting in the identification of 14 genes (Fig. 5a). These 14 genes were systematically compared through TCGA and CGGA glioma databases to assess their significance in glioma expression, molecular subtypes, survival relevance, and to conduct GSEA enrichment analysis for ferroptosis association. There is one protein REST met the specified criteria. Comparative analysis between GTEX and TCGA glioma databases revealed significantly elevated expression of REST in gliomas (Fig. 5b). TCGA and CGGA databases demonstrated an increased expression of REST with higher WHO grades and higher expression in IDH wild-type compared to IDH mutant types (Fig. 5c–f). Survival analysis from TCGA and CGGA databases indicated that patients with high REST expression had shorter survival times (Fig. 5g, h). GSEA enrichment analysis demonstrated a strong correlation between REST and ferroptosis (Fig. 5i, j). These results suggest that REST is a likely binding target of erianin in inducing ferroptosis in GBM cells. Molecular docking between erianin and REST further supported their binding affinity (Fig. 5k).

REST, an inhibitory transcription factor, has been reported with both tumor promotor and tumor suppressor effects. To validate our bioinformatics results, next, a small-molecule pull-down assay was conducted. U87R and GSCm01R cell lysates were incubated with biotin-labeled erianin in the absence or presence of a tenfold excess of unlabeled erianin, followed by pull-down with streptavidin-agarose. The results showed that on band three, REST was significantly bound to and pulled down by biotin-labeled erianin in both U87R and GSCm01R cells. On band four, this binding was competed away by high concentrations of unlabeled erianin (ten times the amount of biotin-labeled erianin) (Fig. 5l), indicating that the REST protein that bound biotin-erianin also bound unlabeled erianin, thus confirming that erianin targets and binds to REST.

To gain a deeper understanding of how erianin impacts REST, we conducted western blotting and qPCR experiments to detect REST expression changes in U87, U87R, GSCm01, and GSCm01R cells after erianin treatment. Our results revealed that, regardless of whether the glioblastoma cells were sensitive or resistant to TMZ, erianin did not alter REST expression (Fig. 5m–o). Concurrently, we observed higher REST expression in TMZ-resistant GBM cells U87R and GSCm01R compared to U87 and GSCm01, suggesting a potential association between REST and GBM TMZ resistance.

We further examined REST expression in 70 glioma tissue specimens from our glioma bank. qPCR revealed significantly higher REST expression in glioma compared to normal brain tissue in our cohort (Fig. 5q), with the highest expression in GBM correlating with increased WHO grades (Fig. 5r). Western blotting (Fig. 5s) and immunohistochemistry (Fig. 5p) confirmed these findings. In summary, these results suggest that REST is the target of erianin in regulating gliomas, overexpressed in glioma and correlating with poor patient prognosis.

### Erianin promotes TMZ sensitivity in TMZ-resistant GSCs through REST

**Fig. 6:**
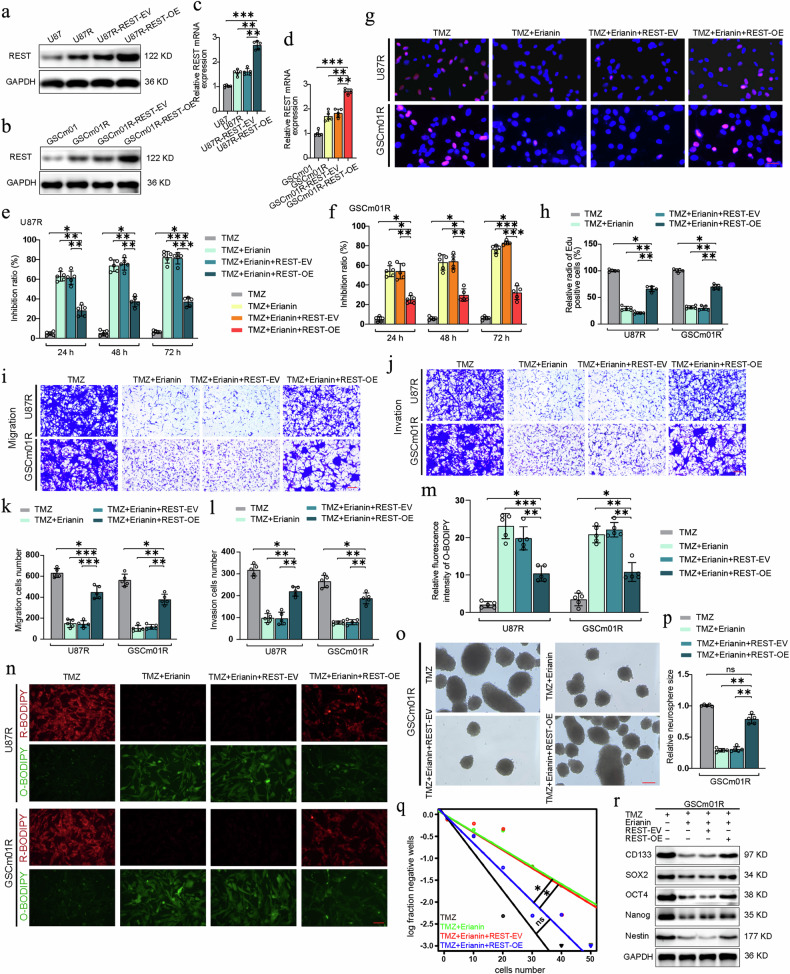
Erianin promotes TMZ sensitivity in TMZ-resistant GSCs through REST. **a**–**d** Western blotting and qPCR assays showing REST protein (**a**, **b**) and mRNA expression (**c**, **d**) in normal, TMZ resistant, and REST overexpressed U87R and GSCm01R. **e**, **f** Cell viability measured with CCK-8 in REST overexpressed U87R and GSCm01R after combined treatment with TMZ and erianin. **g**, **h** Representative EdU assay showing proliferation and quantitative analysis in REST overexpressed U87R and GSCm01R after combined treatment with erianin and TMZ. Scale bar = 50 μm. **i**, **k** Migration assays of REST overexpressed U87R and GSCm01R using a Transwell 24-well plate without extracellular matrix gel after combined treatment. Scale bar = 50 μm. **j**, **l** Invasion assays of REST overexpressed U87R and GSCm01R using a Transwell 24-well plate with extracellular matrix gel after combined treatment. Scale bar = 50 μm. **m**, **n** ferroptosis detection by BODIPY (581/591) C11 probe in U87R and GSCm01R with combined treatment after REST overexpression and quantification of relative fluorescence intensity by Image J. Scale bar = 100 μm. **o**, **p** NSFA revealing the size of neurospheres and quantitative analysis of REST overexpressed GSCm01 after combined treatment with TMZ and erianin. Scale bar = 50 μm. **q** ELDA showing the neurosphere-forming ability of REST overexpressed GSCm01R after combined treatment with TMZ and erianin. **r** Western blotting of stemness marker in REST overexpressed GSCm01R after combined treatment with TMZ and erianin. All data are shown as the mean ± SD (five independent experiments). *p < 0.05; **p < 0.01; ***p < 0.001; ns no significance.

Although erianin treatment did not affect the expression level of REST, we speculate that since erianin binds to REST, it may inhibit the binding of REST to promoter regions, thereby suppressing its transcriptional repression function. First of all, to validate whether erianin exert it is anticancer effects and induces ferroptosis to promotes TMZ sensitivity through REST, we initially designed REST overexpression and knockdown in U87R and GSCm01R cells, and conducted phenotyping experiments to observe whether changes in REST can mediate the anticancer effect of erianin. To begin with, the efficiency of REST overexpression was verified through western blotting and qPCR detection (Fig. 6a–d). Subsequent CCK-8 assays revealed that co-treatment with erianin and TMZ significantly suppressed the viability of U87R and GSCm01R cells. However, when REST was overexpressed in these cells, the inhibitory effect on cell viability and proliferation was less pronounced (Fig. 6e, f), EdU assays confirmed that REST overexpression reversed the reduction in EdU positive cell rates induced by erianin and TMZ in U87R and GSCm01R (Fig. 6g, h). Conversely, when REST was knocked down, the inhibitory effect of erianin on the viability and proliferation of U87R and GSCm01R cells was further enhanced (Supplementary Fig. 2a–d). Subsequently, transwell assays further confirmed that erianin combined with TMZ significantly reduced the number of migrated and invaded U87R and GSCm01R cells. However, after REST overexpression, the number of migrated and invaded cells increased (Fig. 6i–l) and after REST knockdown the number of migrated and invaded cells further decreased compared to the control group (Supplementary Fig. 2e–h). Additionally, using the BODIPY (581/591) C11 probe, we examined the ferroptosis markers in U87R and GSCm01R after REST overexpression. The results indicated a decrease in lipid peroxidation levels after erianin combined with TMZ, and after REST overexpression, the lipid peroxidation levels increased, indicating a reduction in ferroptosis in U87R and GSCm01R (Fig. 6m, n), But after REST knockdown, we observed much higher lipid peroxidation levels in these cells, indicating that REST knockdown promotes ferroptosis in U87R and GSCm01R (Supplementary Fig. 2l, m). Furthermore, NSFA and ELDA experiments revealed that erianin combination with TMZ, reduced the relative size and number of neurospheres in GSCm01R, and REST overexpression re-increased their size and number, while REST knockdown inhibits its sphere-forming ability (Fig. 6o–q, Supplementary Fig. 2i–k). Finally, western blotting detected changes in stemness markers, with REST overexpression counteracting the inhibitory effects of erianin on GSCm01R (Fig. 6r). In conclusion, our results suggest that REST overexpression can reverse the inhibitory effects of erianin, and conversely, REST knockdown enhances the inhibitory effects of erianin, indicating that REST is the target of erianin in regulating gliomas and TMZ-resistant gliomas. Moreover, combination of erianin and TMZ treatment promotes TMZ sensitivity through REST-regulated ferroptosis in U87R and GSCm01R.

### REST Promotes SLC40A1 expression in TMZ-resistant GSCs via inhibiting LRSAM1-mediated SLC40A1 ubiquitination and degradation

**Fig. 7:**
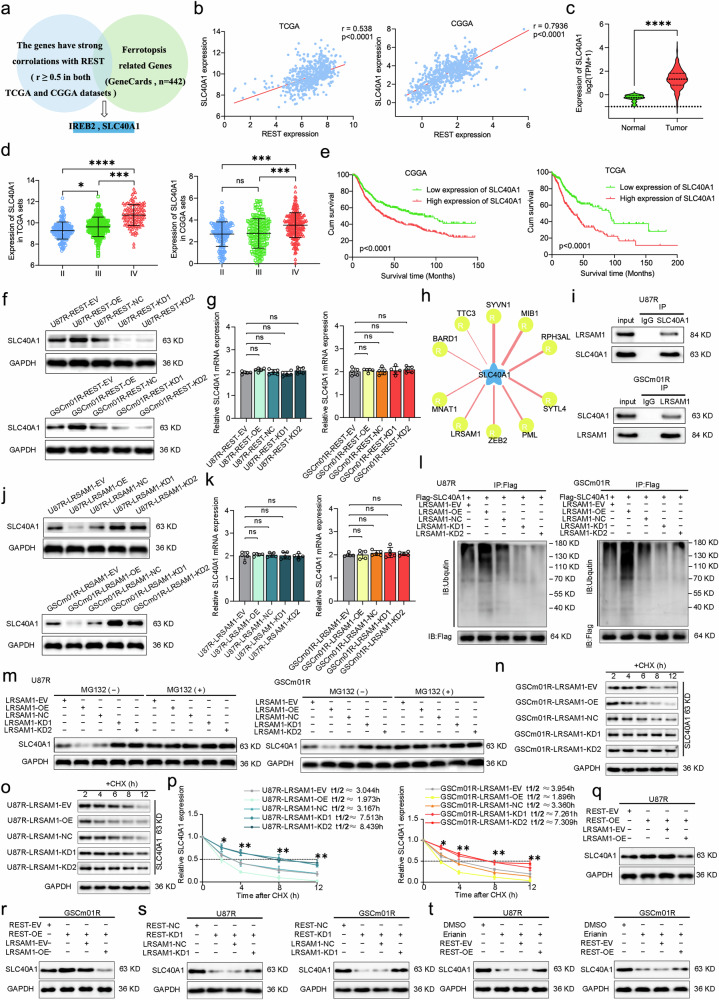
REST Promotes SLC40A1 expression in TMZ-resistant GSCs via inhibiting LRSAM1 mediated SLC40A1 ubiquitination and degradation. **a** Venn chart of REST downstream target screening. **b** Correlation analysis of REST and SLC40A1 based on TCGA and CGGA datasets. **c** SLC40A1 expression was correlated with NBT and glioma based on GTEX and TCGA datasets. **d** SLC40A1 expression was correlated with glioma WHO grades based on TCGA and CGGA datasets. **e** Kaplan–Meier survival curve for all glioma patients with high and low SLC40A1 expression based on TCGA and CGGA datasets. Western blotting and qPCR assays showing SLC40A1 protein (**f**) and mRNA expression (**g**) after REST overexpression and knockdown. **h** Using UbiBrowser 2.0 found the known E3 ubiquitination ligase of SLC40A1. **i** Co-IP assays showed the interaction of LRSAM1 and SLC40A1 in U87R and GSCm01R. Western blotting and qPCR assays showing SLC40A1 protein (**j**) and mRNA expression (**k**) after LRSAM1 overexpression and knockdown. **l** Ubiquitination assays showed the SLC40A1 ubiquitination levels in U87R and GSCm01R followed by LRSAM1 knockdown or overexpressed. **m** The LRSAM1 knockdown or overexpressed U87R and GSCm01R were treated with or without MG132 (50 μM) for 6 hours, and SLC40A1 expression was detected by western blotting. The LRSAM1 knockdown or overexpressed U87R and GSCm01R were treated with CHX (50 μg/ml) and the expression of SLC40A1 protein was detected by western blotting (**n**, **o**) and the half-life time (*t*_1/2_) was quantitative analysis (**p**). **q**, **r** SLC40A1 expression was detected in U87R and GSCm01R by western blotting after REST overexpressing combined with LRSAM1 overexpressing. **s** SLC40A1 expression was detected in U87R and GSCm01R by western blotting after REST silencing combined with LRSAM1 silencing. **t** SLC40A1 expression was detected by western blotting after Erianin treatment combined with REST overexpressed U87R and GSCm01R. All data are shown as the mean ± SD (five independent experiments). *p < 0.05; **p < 0.01; ***p < 0.001; ns no significance.

After modulating REST expression, we found that erianin indeed inhibiting TMZ-resistant gliomas and induces ferroptosis through REST. So next, to investigate potential downstream mechanisms of erianin-induced ferroptosis in TMZ-resistant GSCs, we sought genes strongly correlated with REST in TCGA and CGGA databases. Next, we intersect these genes with the ferroptosis pathway gene set from GeneCards (Table S8), and it revealed two possible genes, SLC40A1 and IREB2 (Fig. 7a). Subsequent analyses prioritized SLC40A1 over IREB2, since IREB2 has no expression or survival significance in glioma. Conversely, TCGA and CGGA database analyses demonstrated a strong correlation between SLC40A1 and REST (Fig. 7b). Meanwhile, GTEX and TCGA database comparisons showed significant elevated SLC40A1 expression in glioma compared to normal brain tissue, with increased expression correlating with higher WHO grading (Fig. 7c, d). Survival analysis indicated that patients with high SLC40A1 expression had shorter survival times (Fig. 7e), suggesting its association with glioma malignancy and relevance to erianin and REST-related ferroptosis.

SLC40A1, also known as Ferroportin (FPN), is a membrane transport protein responsible for intracellular ferrous iron efflux [21]. It plays a crucial role in inhibiting ferroptosis by preventing the accumulation of intracellular ferrous ions, which induce lipid peroxidation through the Fenton reaction [13]. Therefore, when SLC40A1 levels increase in GBM cells, it may exert pro-cancer effect by inhibiting ferroptosis [22]. To validate our findings, we designed REST overexpression and knockdown in U87R and GSCm01R cells. Western blotting revealed that REST overexpression significantly increased SLC40A1 expression, while REST knockdown decreased SLC40A1 expression (Fig. 7f). However, qPCR results surprisingly showed minimal changes in SLC40A1 mRNA expression in U87R and GSCm01R cells after REST overexpression or knockdown compared to the control group (Fig. 7g). This suggests that REST regulates SLC40A1 at the protein level rather than the transcriptional level.

Through this discovery, we investigated the protein degradation pathway of SLC40A1. Ubiquitination is a common protein modification closely related to protein degradation. Most functional proteins in cells are degraded through the ubiquitin-proteasome system. After systematically compared through all E3 ubiquitination ligase of SLC40A1(Fig. 7h). We found a strong negative correlation between REST and one of the E3 ubiquitination ligase of SLC40A1, namely LRSAM1(Supplementary Fig. 3a, b).

LRSAM1 belongs to the RING Finger E3 ubiquitination ligase, which is itself low expressed in gliomas and associated with better patient prognosis (Supplementary Fig. 3c–g). Based on our findings, we hypothesize that REST, as an inhibitory transcription factor, suppresses the transcription of LRSAM1, thereby inhibiting LRSAM1 mediated ubiquitination of SLC40A1, ultimately suppressing ferroptosis in TMZ-resistant GSCs. We detected the expression of LRSAM1 after REST overexpression and knockdown using qPCR and western blotting. As expected, LRSAM1 expression was downregulated after REST overexpression and upregulated after REST knockdown (Supplementary Fig. 3h–k). Confirming the inhibitory transcriptional regulatory effect of REST on LRSAM1. To further validate the transcriptional regulation of LRSAM1 by REST, the promoter sequence region of LRSAM1 was excavated by NCBI and nucleotides including the LRSAM1 promoter (human LRSAM1 site −2000 to 100) were used to predict potential transcription factor binding sites. Potential binding sites for transcription factors to the LRSAM1 promoter region were predicted via Jaspar database (Supplementary Fig. 3l). Subsequently, we analyzed the potential REST transcription binding sites in the LRSAM1 promoter region and designed two mutated nucleotide fragments within the REST binding sites (Supplementary Fig. 3m) for luciferase reporter gene assays. The results showed that REST overexpression significantly reduced luciferase activity in U87R and GSCm01R with the LRSAM1-wt construct, while REST knockdown increased luciferase activity in these cells (Supplementary Fig. 3n, o). Additionally, ChIP assays demonstrated that REST overexpression enhanced the enrichment of LRSAM1 in U87R and GSCm01R in response to anti-REST treatment, while REST silencing decreased this enrichment (Supplementary Fig. 3p, q). Taken together, REST exhibits inhibitory transcriptional regulatory functions on LRSAM1 expression.

To confirm the regulatory role of LRSAM1 in SLC40A1 ubiquitination and degradation, we design LRSAM1 overexpression and knockdown in U87R and GSCm01R. Consistent with the results of ubiquitination degradation regulation, LRSAM1 overexpression did not alter SLC40A1 mRNA levels but significantly decreased SLC40A1 protein levels, while LRSAM1 knockdown did not change SLC40A1 mRNA levels but increased its protein levels (Fig. 7j, k). Co-IP results demonstrated the interaction between LRSAM1 and SLC40A1 in U87R and GSCm01R cells (Fig. 7i). Subsequent ubiquitination assays revealed that LRSAM1 overexpression significantly enhanced the ubiquitination of SLC40A1, while LRSAM1 knockdown resulted in lower levels of ubiquitinated SLC40A1 (Fig. 7l). Furthermore, the addition of the proteasome inhibitor MG132 rescued the increased degradation of SLC40A1 caused by LRSAM1 overexpression in U87R and GSCm01R. Conversely, MG132 treatment further increased the accumulation of SLC40A1 in U87R and GSCm01R with LRSAM1 knockdown (Fig. 7m). CHX pulse-chase assay confirmed that LRSAM1 overexpression significantly shortened the half-life of SLC40A1 in U87R and GSCm01R, while LRSAM1 knockdown markedly extended the half-life of SLC40A1 (Fig. 7n–q). In summary, SLC40A1 ubiquitination and degradation depend on the E3 ubiquitin ligase LRSAM1, and elevated LRSAM1 levels promote the ubiquitination and degradation of SLC40A1.

Finally, we performed various western blot analyses. REST knockdown decreased SLC40A1 protein levels, but this effect was reversed by LRSAM1 knockdown (Fig. 7q, r). Conversely, REST overexpression increased SLC40A1 protein levels, which could be reversed by LRSAM1 overexpression (Fig. 7s). Lastly, erianin treatment decreased SLC40A1 levels, but this effect was counteracted by REST overexpression (Fig. 7t).

In conclusion, REST promotes SLC40A1 expression in TMZ-resistant GSCs by inhibiting LRSAM1-mediated ubiquitination and degradation, ultimately contributing to erianin-induced ferroptosis in TMZ-resistant GSCs.

### Erianin promotes TMZ sensitivities in TMZ-resistant GSCs via mediating SLC40A1 Ubiquitination through REST/LRSAM1

We further investigated how the REST/LRSAMI pathway and SLC40A1 ubiquitination levels changed in TMZ-resistant GSCs before and after erianin administration and validated the role of erianin in promoting TMZ sensitivity in TMZ-resistant GSCs through ferroptosis by mediating SLC40A1 ubiquitination. Firstly, after treating with erianin, we performed a series of experiments to assess the changes in REST/LRSAM1 cascade signaling before and after erianin treatment. ChIP assays demonstrated that erianin treatment significantly decreased the enrichment of LRSAM1 in U87R and GSCm01R in response to anti-REST treatment, and when U87R and GSCm01R with REST overexpression were treated with erianin, this decreased enrichment has recovered somewhat (Supplementary Fig. 4a). Luciferase reporter gene assays demonstrated that erianin treatment significantly increased luciferase activity in U87R and GSCm01R with the LRSAM1-wt construct, while REST overexpression decreased luciferase activity in these cells after erianin treatment (Supplementary Fig. 4b, c). After that, we detected the expression of LRSAM1 after erianin treatment using qPCR and western blotting. The results show that LRSAM1 expression was upregulated after erianin treatment and downregulated after combination with REST overexpression (Supplementary Fig. 4d, e).

Moving forward, to investigate how the ubiquitination levels of SLC40A1 altered in TMZ-resistant GSCs prior to and following erianin treatment, we proceeded with ubiquitination assays, CHX pulse-chase assays, and MG132 rescue assays. ubiquitination assays revealed that erianin significantly enhanced the ubiquitination of SLC40A1 (Supplementary Fig. 5a, b). Furthermore, the addition of the proteasome inhibitor MG132 rescued the increased degradation of SLC40A1 caused by erianin treatment in U87R and GSCm01R (Supplementary Fig. 5c, d). CHX pulse-chase assay confirmed that erianin significantly shortened the half-life of SLC40A1 in U87R and GSCm01R (Supplementary Fig. 5e–h), All these results indicated an increase in the ubiquitination level of SLC40A1 after the administration of erianin.

Subsequently, we conducted phenotyping experiments in U87R and GSCm01R cells after overexpressing SLC40A1, followed by combined treatment with erianin and TMZ. CCK-8 assays revealed that, in SLC40A1-overexpressing U87R and GSCm01R cells, the inhibitory effect on cell viability by erianin in combination with TMZ was significantly reduced (Supplementary Fig. 6a, b). EdU assays further indicated that SLC40A1 overexpression reversed the decrease in EdU positive cell rates induced by combined treatment with erianin and TMZ in U87R and GSCm01R cells (Supplementary Fig. 6c, d). This suggests that SLC40A1 overexpression can counteract the inhibitory effects of erianin and TMZ on cell viability and proliferation. transwell assays confirmed that SLC40A1 overexpression increased the migration and invasion of U87R and GSCm01R cells after treatment with erianin and TMZ (Supplementary Fig. 6e–h). Additionally, BODIPY (581/591) C11 probe assays demonstrated that SLC40A1 overexpression reduced ferroptosis in U87R and GSCm01R cells compared to the control group (Supplementary Fig. 6i, j). NSFA and ELDA assays revealed that SLC40A1 overexpression enhanced the relative size and number of neurospheres in GSCm01R after treatment with erianin and TMZ (Supplementary Fig. 6k–m). Western blotting further confirmed the increase in stemness marker expression in SLC40A1-overexpressing GSCm01R cells after combined treatment with erianin and TMZ (Supplementary Fig. 6n). Considering the biological function of SLC40A1 in transporting intracellular ferrous iron to the extracellular space, we measured intracellular ferrous iron levels in U87R and GSCm01R cells after treatment with erianin and TMZ. The results showed an increase in intracellular ferrous iron levels after treatment, but SLC40A1 overexpression reversed this effect (Supplementary Fig. 6o), indicating that erianin and TMZ treatment inhibits the function of SLC40A1. In conclusion, erianin promotes the TMZ sensitivities of TMZ-resistant GSCs by mediating SLC40A1 ubiquitination and degradation, providing evidence for its potential therapeutic role in GBM.

### Erianin inhibits tumor formation of TMZ-resistant GSCs in vivo

**Fig. 8:**
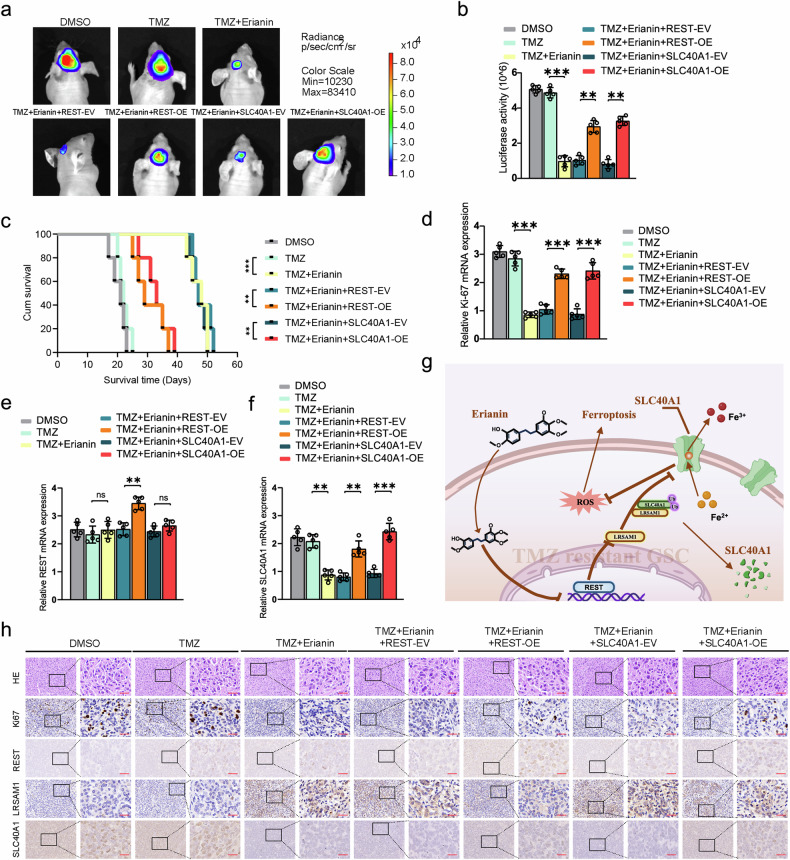
Erianin inhibits tumor formation of TMZ-resistant GSCs in vivo. Bioluminescence imaging (**a**) and quantification (**b**) of luciferase intensity in GSCm01R xenografted nude mice treated with multiple groups (DMSO, TMZ, TMZ + Erianin, TMZ + Erianin + REST-EV, TMZ + Erianin + REST-OE, TMZ + Erianin + SLC40A1-EV, TMZ + Erianin + SLC40A1-OE). **c** Kaplan–Meier survival analysis illustrates the survival times of GSCm01R xenografted nude mice of different mentioned treatment groups. **d**–**f** qPCR assays showing the expression of REST, SLC40A1, and Ki-67 in the above-mentioned multiple groups. **g** Schematic diagram illustrating erianin inducing ferroptosis in TMZ-resistant GSCs through REST/LRSAM1 mediated ubiquitination degradation of SLC40A1. **h** Representative H&E and immunohistochemical images showed the staining and expression of REST, LRSAM1, SLC40A1 and Ki-67 of the multiple groups mentioned above. Scale bar = 50 μm. All data are shown as the mean ± SD (five independent experiments). *p < 0.05; **p < 0.01; ***p < 0.001; ns, no significance.

To demonstrate the inhibitory effect of erianin in combination with TMZ on the tumor formation ability of TMZ-resistant GSCs in vivo, we conducted xenograft experiments using GSCm01R. Vivo fluorescence imaging showed a significant reduction in tumor volume and size in mice treated with erianin in combination with TMZ, while GSCm01R cells overexpressing REST or SLC40A1 exhibited increased tumor volume and size (Fig. 8a, b). Kaplan–Meier survival analysis revealed an extended median survival time in the erianin and TMZ treatment group compared to the DMSO and TMZ groups, while the median survival time was shortened in the groups overexpressing REST or SLC40A1 (Fig. 8c). qPCR assays on Ki-67, REST and SLC40A1 expression levels showed a significant reduction in Ki-67 and SLC40A1 expression with erianin and TMZ co-treatment, but after REST or SLC40A1 overexpression reversed or even upregulated the expression of Ki-67 and SLC40A1 (Fig. 8d, f), on the contrary, the expression of REST did not change with co-treatment (Fig. 8e), suggest erianin inhibit REST transcriptional repression function, does not affect its expression level. In our study, the ferroptosis pathway in GSCs, mediated by REST, LRSAM1, and SLC40A1, was morphologically identified through immunohistochemical staining of orthotopic xenograft tumor specimens. After co-treatment with erianin and TMZ, the IHC staining intensity of Ki-67 and SLC40A1 decreased, while the IHC staining intensity of LRSAM1 increased. Meanwhile, overexpression of REST or SLC40A1 could reverse this effect. And as expected, the IHC staining intensity of REST did not change after the co-treatment (Fig. 8h). In summary, the schematic diagram illustrates that erianin promotes TMZ sensitivities in TMZ-resistant GSCs and induces ferroptosis through REST downregulation and SLC40A1 Ubiquitination and degradation (Fig. 8g).

## Discussion

In recent years, anti-tumor drugs derived from plants have garnered attention due to their unique physiological activities, superior efficacy, lower toxicity, natural structural alignment with biological targets, and multifaceted functionality. And furthermore, their multi-targeting properties hold the potential to overcome drug resistance [23]. Dendrobium chrysotoxum Lindl, is a precious traditional Chinese herbal medicine with various medicinal values and biological activities, and erianin is the important active ingredient in it. While several studies have reported the anticancer activity of erianin on various cancer [24], yet its effects on glioma remain unclear. And moreover, erianin has demonstrated minimal damage to normal cells in previous studies [25–27], and a study revealed no significant damage to internal organs in animals treated with erianin during animal experiments [11], indicating minimal side effects on normal cells and organs of the body. Apart from the side effects, the permeability of the blood–brain barrier is also crucial in treating intracranial lesions. Erianin is a small-molecule compound, has been predicted to have a relatively high blood-brain barrier permeability in a new study [28], which also conducted an ADMET (Absorption, Distribution, Metabolism, Excretion, and Toxicity) prediction, yielding positive results. As a potential drug, erianin exhibits satisfactory ADMET characteristics. However, erianin has its shortcomings, such as poor water solubility. Additionally, pharmacokinetic studies in animals indicate that erianin experiences a rapid decline in blood concentration after entering the body, with a short half-life period and low bioavailability [29, 30]. In response, erianin derivatives have been developed to enhance its water solubility [31–33]. And future advancements in carrier material science are anticipated to address and improve these limitations.

TMZ, an alkylating agent, has clear anti-tumor activity as a monotherapy in treating gliomas, particularly effective in gliomas with low expression of MGMT (methylguanine-DNA methyltransferase) [34]. As a standardized chemotherapeutic drug for gliomas, it can improve survival by approximately 2.5 months in clinical treatment. The limitation of its efficacy lies in the inherent or mutation-acquired TMZ resistance of the patient’s tumor subtype [6]. Consequently, addressing TMZ resistance and exploring combination therapies that are more effective against TMZ-resistant gliomas have become critical challenges in clinical treatment of gliomas. TMZ exerts its anticancer activity by inducing tumor cell apoptosis through DNA damage. Ferroptosis, as a novel form of cell death distinct from apoptosis, may bypass the anticancer mechanisms of TMZ and represents a potential therapeutic strategy for TMZ-resistant gliomas [35]. Studies have shown that cancer cells resistant to traditional radiation and chemotherapy are more prone to undergo ferroptosis [36, 37]. Buccarelli et al. reported that inhibition of autophagy in GBM induces ferroptosis, thereby increasing GSCs sensitivity to TMZ [38]. Chen et al. demonstrated that using the ferroptosis inducer Erastin, by inhibiting the function of xCT and cystathionine-γ-lyase, sensitizes GSCs to TMZ [39]. Therefore, new drugs targeting ferroptosis in glioma cells hold promise as a prospective treatment strategy for patients with TMZ-resistant gliomas.

In this study, we report for the first time that erianin inhibits the viability of GSCs, including TMZ-resistant GSCs, and that the combination of erianin with TMZ promotes the sensitivity of TMZ in TMZ-resistant GSCs. Subsequent experiments revealed that the underlying mechanism involves inducing ferroptosis in TMZ-resistant GSCs. Therefore, to determine how erianin induces ferroptosis in TMZ-resistant GSCs, we explored through network pharmacology and found that erianin likely targets a transcription factor, REST. The importance of transcription factors in the development and progression of tumors is well recognized, with many cancers exhibiting abnormal expression and function. Overexpression and mutations of some transcription factors can lead to the onset and worsening of tumors. REST (RE1-silencing transcription factor), also known as neuron-restrictive silencer factor or NRSF, is a Kruppel-type zinc finger transcription factor with 8 zinc fingers and two repression domains located at the N and C termini [40]. REST is a master repressor of neuronal gene expression and neuronal programs in non-neuronal lineages [41]. Previous studies have reported REST as both a tumor promoter and tumor suppressor [42, 43], indicating that REST regulation might have significant physiological and pathological consequences. However, the pathways controlling REST are not yet fully elucidated. Here, we demonstrate that REST can be targeted and bound by erianin, inhibiting its transcription factor function, leading to a reduced ability to transcriptionally repress the E3 ubiquitin ligase LRSAM1.

E3 ubiquitin ligases are part of the ubiquitin-proteasome system (UPS), a multi-component system responsible for the degradation of proteins within the cell. By regulating the degradation of most proteins, UPS is involved in various cellular processes such as cell growth and differentiation, DNA replication and repair, metabolism, and immune response. Malignant tumor cells require an efficient protein homeostasis to sustain rapid proliferation, and maintaining protein homeostasis is a key metabolic event in controlling the proliferation of malignant tumors. Thus, the role of UPS in malignancies holds significant potential for exploration, although its precise regulatory mechanisms in tumors are not yet fully understood. In the context of malignant tumors, the functions of a series of E3 ubiquitin ligases in gliomas have been reported. For instance, our previous research found that circRNF10 can competitively bind to the E3 ubiquitin ligase MKRN3 and block its ubiquitin ligase activity, thereby enhancing the expression of ZBTB48. ZBTB48 promotes the malignant progression of gliomas by inducing GSCs to evade ferroptosis [19]. In contrast, the E3 ligase TRIM21 accelerates tumor progression in vitro and in vivo through K63-linked polyubiquitination of β-catenin, leading to its nuclear translocation via the Wnt/β-catenin signaling pathway [44]. This suggests that E3 ligases can act as tumor suppressors or promoter in the malignant progression of gliomas. E3 ubiquitin ligase LRSAM1 (Leucine-rich repeat and sterile alpha motif-containing 1) is a really interesting new gene (RING) class protein that possesses E3 ubiquitin ligase activity with promising applications in UPS [45]. Previous studies have elucidated the relevance of LRSAM1 to brain diseases and its molecular biological mechanisms [46]. In our study, we used UbiBrowser 2.0 (http://ubibrowser.bio-it) in combination with preliminary predictions of protein interactions and further screening through protein-protein interaction experiments. We found that LRSAM1 is most likely the E3 ubiquitin ligase for SLC40A1 in erianin pathway. This indicates that erianin ultimately promotes ferroptosis by affecting the levels of the membrane protein SLC40A1.

It is well known that ferroptosis is a novel form of cell death, characterized by the intricate interplay of iron metabolism, lipid peroxidation, and the antioxidative system within the cell, ultimately marked by the accumulation of ROS and lipid peroxidation of polyunsaturated fatty acids, causing cellular damage. Ferroptosis has also emerged as a new avenue for cancer therapy [47]. In the classical pathways leading to ferroptosis, it is associated with the depletion of glutathione antioxidative capacity [48]. Key molecules in this process are glutathione peroxidase 4 (GPX4) and the cystine-glutamate antiporter (Xc-/xCT), which are crucial for inducing ferroptosis [49, 50]. Besides the classical pathway, intracellular iron metabolism also plays a vital role to inducing ferroptosis. Accumulated Fe^2+^ within the cell leads to a state of peroxidation through the Fenton reaction [13]. Intracellular iron metabolism is mainly controlled by the iron ferritinophagy, which is the iron phagocytosis and reduction of ferritin [51, 52]. as well as the transport of Fe^3+^ from the extracellular to intracellular through transferrin [53], and the transport of Fe^2+^ from the intracellular to extracellular through ferroportin [21]. Ferroportin, also known as SLC40A1, is the only known iron export protein in the cell membrane, making it a key protein in inhibiting ferroptosis and highly relevant to the development and progression of tumors, previous studies have reported the above association [22, 54]. Our research indicates that erianin promotes the ubiquitination of SLC40A1, leading to a decrease in the level of the iron export protein SLC40A1, thereby promoting ferroptosis in GSCs.

In this study, we focused on investigating the inhibitory activity of erianin both in vitro and in vivo on GSCs and TMZ-resistant GSCs. Our findings suggest that erianin may be a novel tumor inhibitor with the potential to induce ferroptosis in regular and TMZ-resistant GBM cells, as well as improve TMZ sensitivity in TMZ-resistant GBM cells. Our data support the potential of erianin as a compound for the treatment of gliomas. To maximize the clinical application potential of erianin, large-scale, multicenter collaborative clinical trials are urgently needed in the future.

### Supplementary information

Supplementary Figure 1

Supplementary Figure 2

Supplementary Figure 3

Supplementary Figure 4

Supplementary Figure 5

Supplementary Figure 6

Supplementary Table S1- S8

Supplementary legends

original data files

aj-checklist

## Data Availability

The analyzed datasets generated during the present study are available from the corresponding author on reasonable request.
