# Supplementary figures and images for: Erianin induces ferroptosis in GSCs via REST/LRSAM1 mediated SLC40A1 ubiquitination to overcome TMZ resistance
Source: Cell Death Dis. 2024 Jul 22;15(7):522. doi: 10.1038/s41419-024-06902-4 (PMC11263394; doi:10.1038/s41419-024-06902-4)

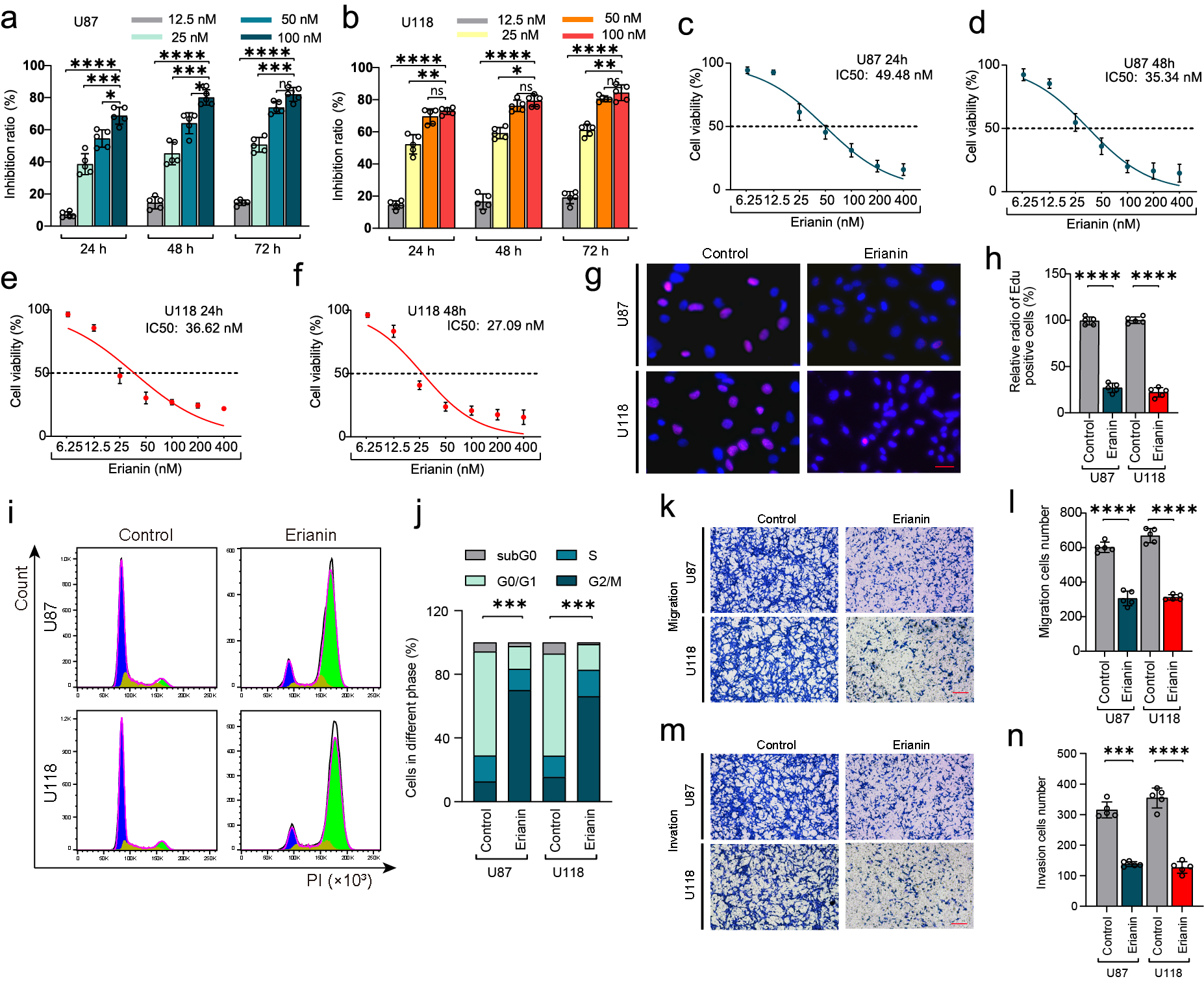

Supplement: Supplementary file 1 — Supplementary Figure 1 [file 41419_2024_6902_MOESM1_ESM.tif]

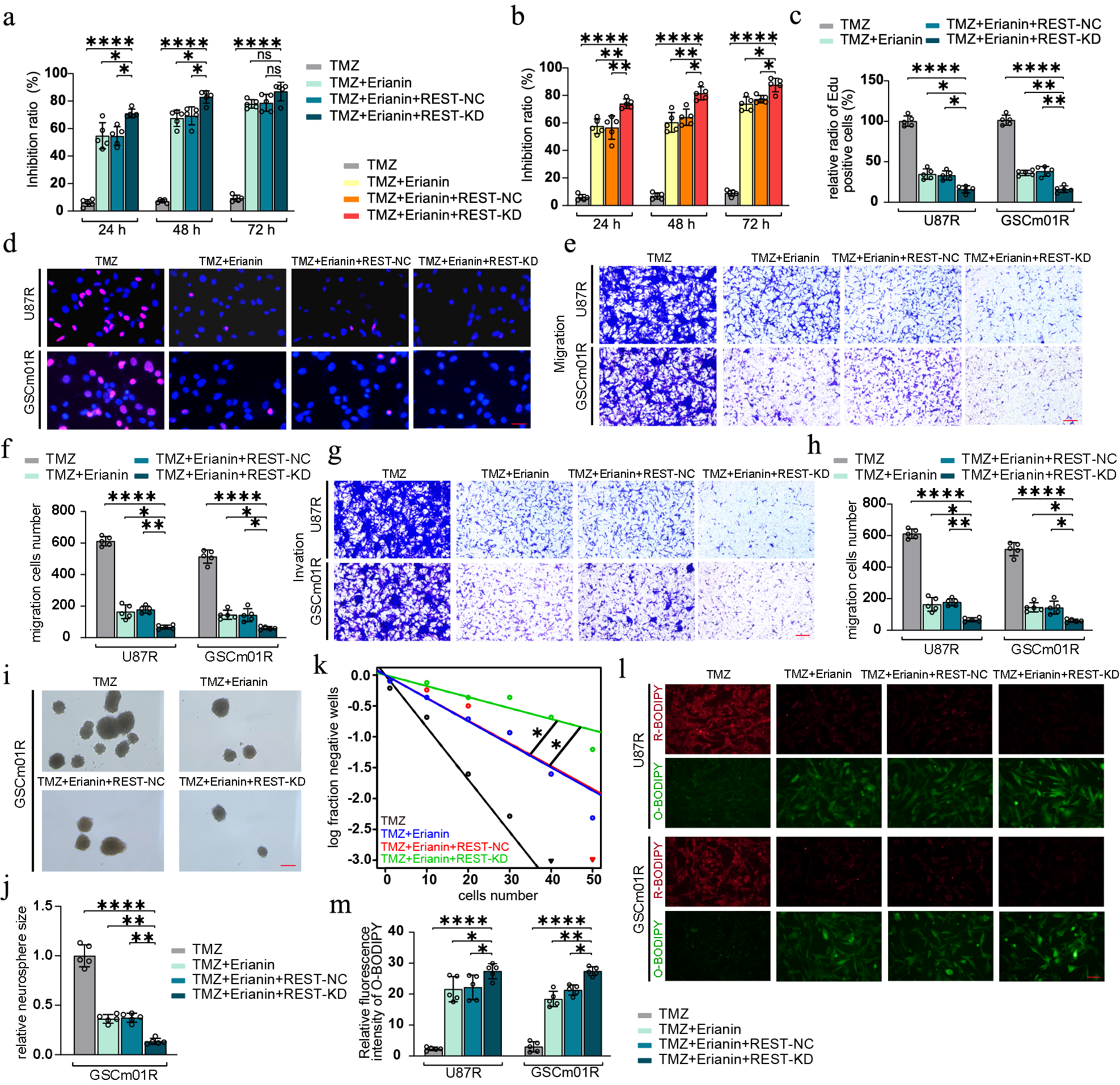

Supplement: Supplementary file 2 — Supplementary Figure 2 [file 41419_2024_6902_MOESM2_ESM.tif]

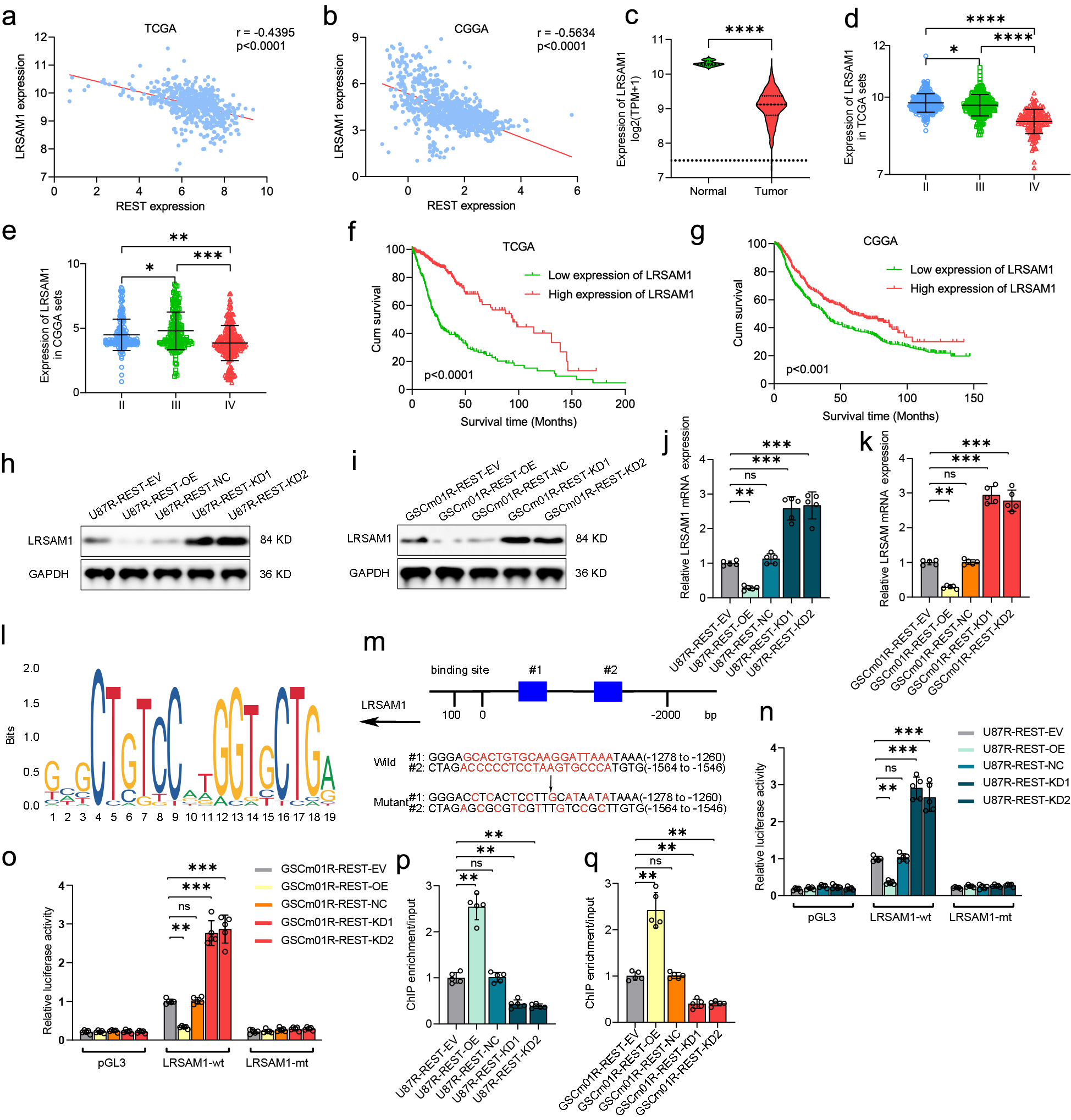

Supplement: Supplementary file 3 — Supplementary Figure 3 [file 41419_2024_6902_MOESM3_ESM.tif]

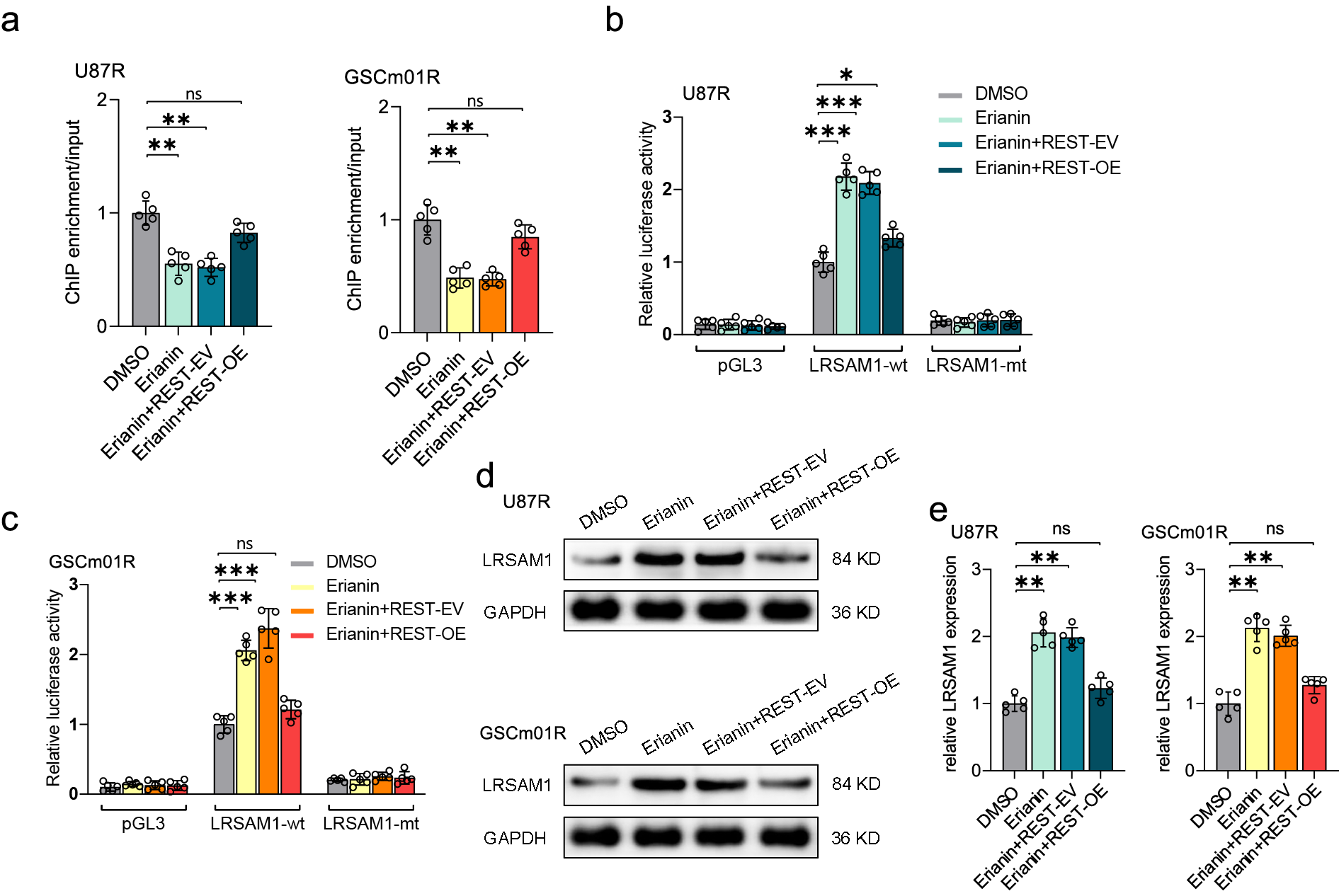

Supplement: Supplementary file 4 — Supplementary Figure 4 [file 41419_2024_6902_MOESM4_ESM.tif]

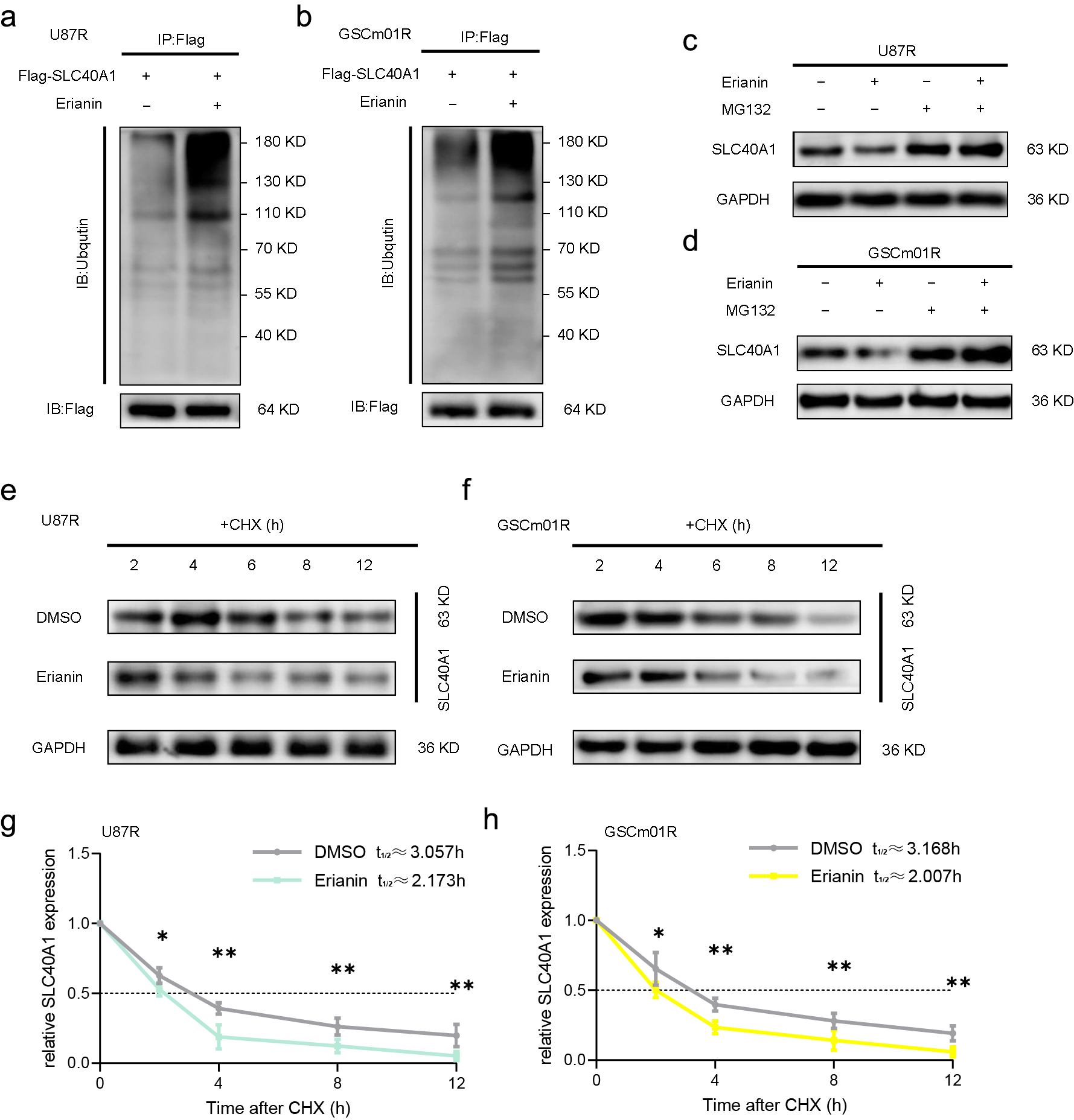

Supplement: Supplementary file 5 — Supplementary Figure 5 [file 41419_2024_6902_MOESM5_ESM.tif]

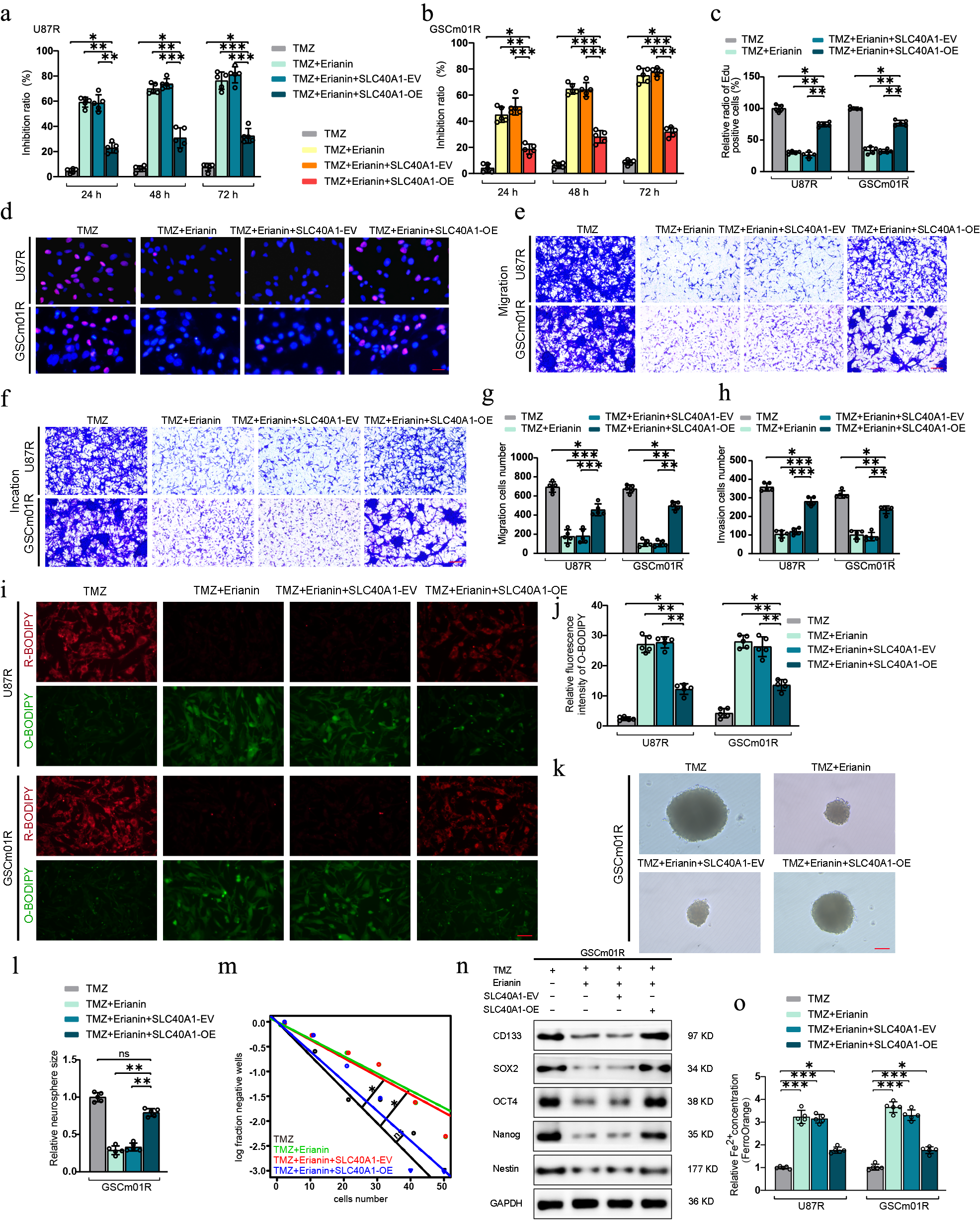

Supplement: Supplementary file 6 — Supplementary Figure 6 [file 41419_2024_6902_MOESM6_ESM.tif]
