# Supplementary material for: Erianin induces ferroptosis in GSCs via REST/LRSAM1 mediated SLC40A1 ubiquitination to overcome TMZ resistance
Source: Cell Death Dis. 2024 Jul 22;15(7):522. doi: 10.1038/s41419-024-06902-4 (PMC11263394; doi:10.1038/s41419-024-06902-4)
Supplement: Supplementary file 8 — Supplementary legends [file 41419_2024_6902_MOESM8_ESM.docx]

**Supplementary Figure 1 Erianin inhibits the malignant progression of GBM.**

**a, b** Cell viability of U87 and U118 after treatment with erianin at different times and concentrations, measured using CCK-8.

**c-f** IC50 of U87 and U118 at 24 and 48 hours after treatment with erianin, measured using CCK-8.

**g, h** Representative EdU assay showing proliferation and quantitative analysis of U87 and U118 after erianin treatment. Scale bar = 50μm.

**i, j** Representative results of cell cycle assay and quantitative analysis of U87 and U118 after erianin treatment.

**k, l** Migration assays of U87 and U118 using a Transwell 24-well plate without extracellular matrix gel, and quantitative analysis after erianin treatment. Scale bar = 50μm.

**m, n** Invasion assays of U87 and U118 using a Transwell 24-well plate with extracellular matrix gel, and quantitative analysis after erianin treatment. Scale bar = 50μm.

All data are shown as the mean ± SD (five independent experiments). *p<0.05; **p<0.01; ***p<0.001; ns, no significance.

**Supplementary Figure 2 Erianin promotes TMZ sensitivity in TMZ resistant GSCs through REST**

**a, b** Cell viability measured with CCK-8 in REST knockdown U87R and GSCm01R after combined treatment with TMZ and erianin.

**c, d** Representative EdU assay showing proliferation and quantitative analysis in REST knockdown U87R and GSCm01R after combined treatment with erianin and TMZ. Scale bar = 50μm.

**e, f** Migration assays of REST knockdown U87R and GSCm01R using a Transwell 24-well plate without extracellular matrix gel after combined treatment. Scale bar = 50μm.

**g, h** Invasion assays of REST knockdown U87R and GSCm01R using a Transwell 24-well plate with extracellular matrix gel after combined treatment. Scale bar = 50μm.

**i, j** NSFA revealing the size of neurospheres and quantitative analysis of REST knockdown GSCm01 after combined treatment with TMZ and erianin. Scale bar = 50μm.

**k** ELDA showing the neurosphere forming ability of REST knockdown GSCm01R after combined treatment with TMZ and erianin.

**l, m** ferroptosis detection by BODIPY (581/591) C11 probe in U87R and GSCm01R with combined treatment after REST knockdown and quantification of relative fluorescence intensity by Image J. Scale bar = 100μm.

All data are shown as the mean ± SD (five independent experiments). *p<0.05; **p<0.01; ***p<0.001; ns, no significance.

**Supplementary Figure 3** **REST Promotes SLC40A1 expression in TMZ resistant GSCs via inhibiting LRSAM1 mediated SLC40A1 ubiquitination and degradation.**

**a, b** Correlation analysis of REST and LRSAM1 based on TCGA and CGGA datasets.

**c** LRSAM1 expression was correlated with NBT and glioma based on GTEX and TCGA datasets. **d, e** LRSAM1 expression was correlated with glioma WHO grades based on TCGA and CGGA datasets.

**f, g** Kaplan-Meier survival analysis for all glioma patients with high and low LRSAM1 expression based on TCGA and CGGA datasets.

**h-k** Western blot and qPCR assays showed LRSAM1 protein expression (h, i) and mRNA expression (j, k) after knockdown and overexpression of REST.

**i** REST binding motif obtained from the Jaspar database.

**m** Shows the two putative REST binding sites and matched mutant sequences in the LRSAM1 promoter region for luciferase reporter assays.

**n, o** The luciferase reporter assays revealed the luciferase promoter activities of LRSAM1 with REST knockdown and or overexpression in U87R and GSCm01R.

**p, q** The ChIP qPCR showed that anti-REST treatment could detect the enrichment difference of LRSAM1 promoter sequence in REST knockdown and or overexpressed U87R and GSCm01R.

All data are shown as mean ± SD (five independent experiments). *p<0.05; **p<0.01; ***p<0.001; ns, no significance.

**Supplementary Figure 4** **Erianin inhibits the transcriptional repression ability of REST on LRSAM1.**

**a** The ChIP qPCR showed that anti-REST treatment could detect the enrichment difference of LRSAM1 promoter sequence after erianin treatment and overexpression of REST.

**b, c** The luciferase reporter assays revealed the luciferase promoter activities of LRSAM1 after erianin treatment and overexpression of REST.

**d, e** Western blot and qPCR assays showed LRSAM1 protein expression (d) and mRNA expression (e) after erianin treatment and overexpression of REST.

All data are shown as mean ± SD (five independent experiments). *p<0.05; **p<0.01; ***p<0.001; ns, no significance.

**Supplementary Figure 5** **Erianin increased the ubiquitination level of SLC40A1.**

**a, b** Ubiquitination assays showed the SLC40A1 ubiquitination levels in U87R and GSCm01R followed by erianin treatment.

**c, d** After erianin treatment, U87R and GSCm01R were treated with or without MG-132 (50μM) for 6 hours, and SLC40A1 expression was detected by western blotting.

**e-h** After erianin treatment, U87R and GSCm01R were treated with CHX (50μg/ml) and the expression of SLC40A1 protein was detected by western blotting (e, f) and the half-life time (t1/2) was quantitative analysis (g, h).

All data are shown as mean ± SD (five independent experiments). *p<0.05; **p<0.01; ***p<0.001; ns, no significance.

**Supplementary Figure 6** **Erianin promotes TMZ sensitivities in TMZ resistant GSCs via mediating SLC40A1 Ubiquitination through REST/LRSAM1.**

**a, b** Cell viability measured with CCK-8 in SLC40A1 overexpressed U87R and GSCm01R after combined treatment with TMZ and erianin.

**c, d** Representative EdU assay showing proliferation and quantitative analysis in SLC40A1 overexpressed U87R and GSCm01R after combined treatment with erianin and TMZ. Scale bar = 50μm.

**e, g** Migration assays of SLC40A1 overexpressed U87R and GSCm01R using a Transwell 24-well plate without extracellular matrix gel after combined treatment. Scale bar = 50μm

**f, h** Invasion assays of SLC40A1 overexpressed U87R and GSCm01R using a Transwell 24-well plate with extracellular matrix gel after combined treatment. Scale bar = 50μm.

**i, j** ferroptosis detection by BODIPY (581/591) C11 probe in U87R and GSCm01R with combined treatment after SLC40A1 overexpression and quantification of relative fluorescence intensity by Image J. Scale bar = 100μm.

**k, l** NSFA revealing the size of neurospheres and quantitative analysis of SLC40A1 overexpressed GSCm01 after combined treatment with TMZ and erianin. Scale bar = 50μm.

**m** ELDA showing the neurosphere forming ability of SLC40A1 overexpressed GSCm01R after combined treatment with TMZ and erianin.

**n** Western blotting of stemness marker in SLC40A1 overexpressed GSCm01R after combined treatment with TMZ and erianin.

**o** Intracellular Fe^2+^ levels in SLC40A1 overexpressed U87R and GSCm01R were measured using the FerroOrange kit after combined treatment with TMZ and erianin.

All data are shown as the mean ± SD (five independent experiments). *p<0.05; **p<0.01; ***p<0.001; ns, no significance.
