# Supplementary material for: Erianin induces ferroptosis in GSCs via REST/LRSAM1 mediated SLC40A1 ubiquitination to overcome TMZ resistance
Source: Cell Death Dis. 2024 Jul 22;15(7):522. doi: 10.1038/s41419-024-06902-4 (PMC11263394; doi:10.1038/s41419-024-06902-4)
Supplement: Supplementary file 9 — original data files [file 41419_2024_6902_MOESM9_ESM.docx]

**Figure 1.t**

**GSCm01 (target at lane1 and lane2)**

CD133

**
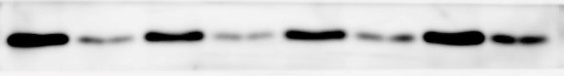
**

SOX2

**
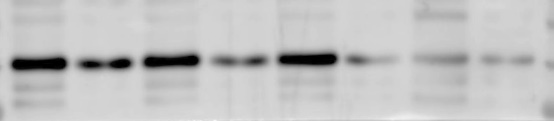
**

OCT4

**
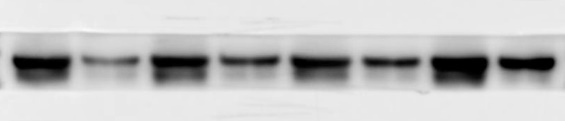
**

Nanog


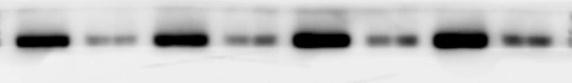


Nestin


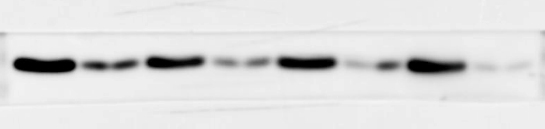


GAPDH

**
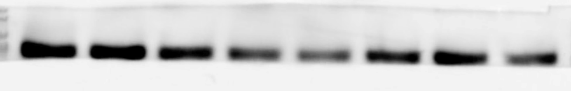
**

**GSCm03 (target at lane3 and lane4)**

CD133

**
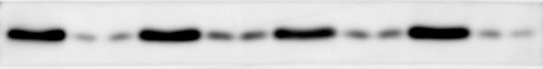
**

SOX2

**
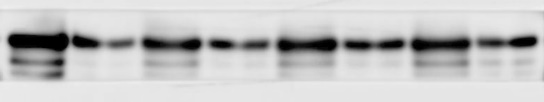
**

OCT4

**
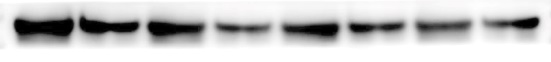
**

Nanog

**
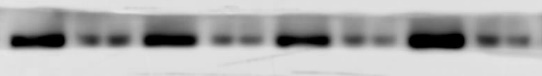
**

Nestin

**
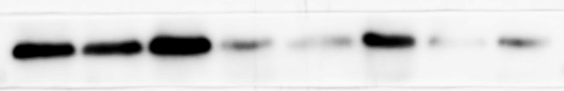
**

GAPDH

**
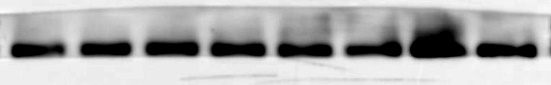
**

**Figure 2.n**

**GSCm01R (target at lane 5,6,7,8)**

CD133

**
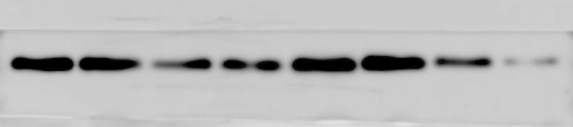
**

SOX2


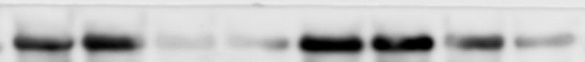


OCT4

**
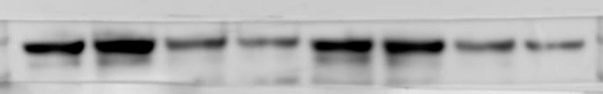
**

Nanog

**
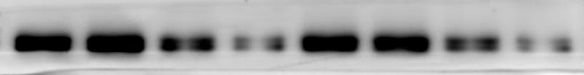
**

Nestin

**
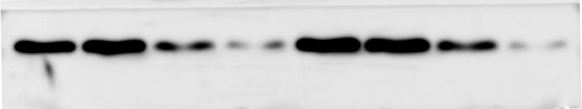
**

GAPDH

**
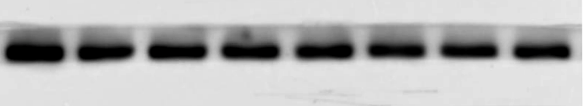
**

**Figure 3.t**

**U87R (target at lane5 and lane6)**

Glutaminase

**

**

GPX4





SLC7A11





SLC40A1


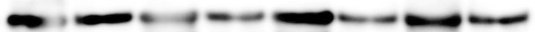


Transferrin


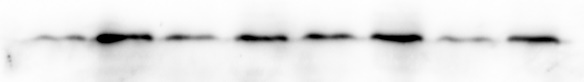


GAPDH


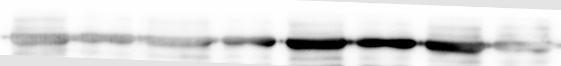


**GSCm01R (target at lane3 and lane4)**

Glutaminase

**

**

GPX4

**
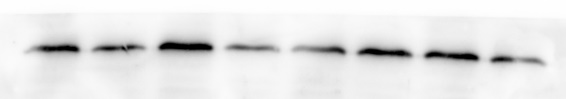
**

SLC7A11

**
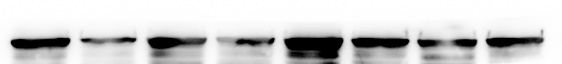
**

SLC40A1


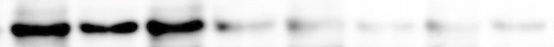


Transferrin





GAPDH

**
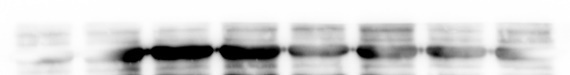
**

**Figure 4.l**

**GSCm01R (target at lane 5,6,7,8)**

CD133

**
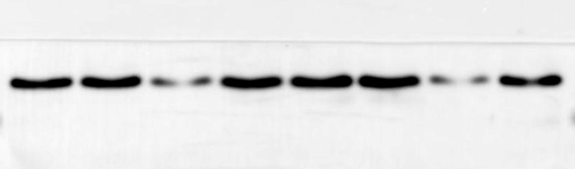
**

SOX2

**
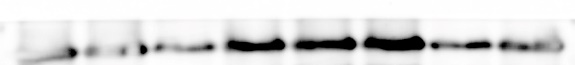
**

OCT4

**
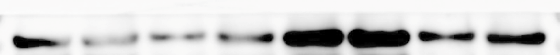
**

Nanog

**
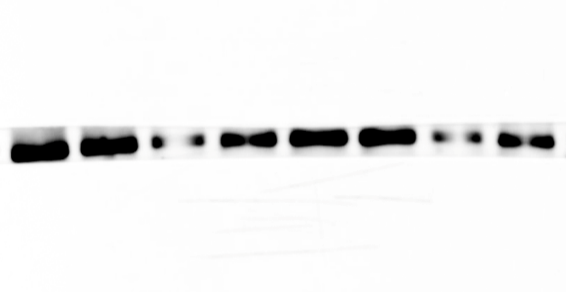
**

Nestin

**
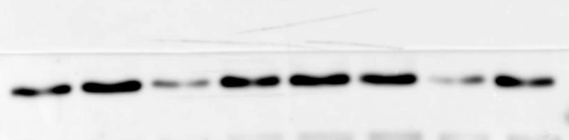
**

GAPDH

**
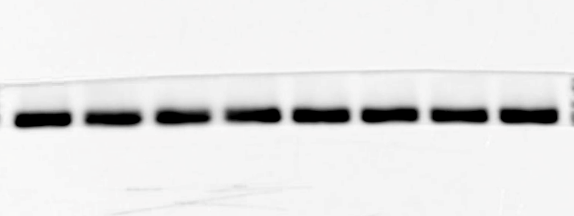
**

**Figure 5.l**

**U87R (target at all lane)**

Biotin


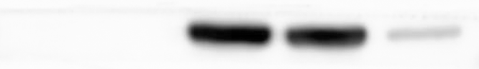


REST

**
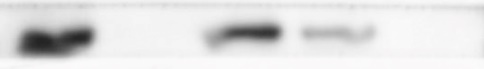
**

**Figure 5.m**

**GSCm01R (target at all lane)**

Biotin

**
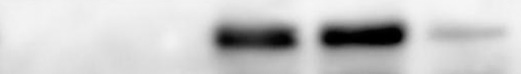
**

REST

**
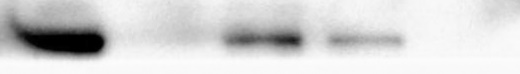
**

**Figure 5.n**

**U87 and U87R (target at lane 3,4,5,6)**

REST

**
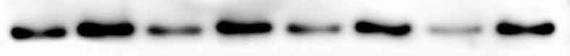
**

GAPDH

**
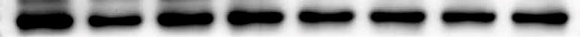
**

**Figure 5.o**

**GSCm01 and GSCm01R (target at lane 5,6,7,8)**

REST


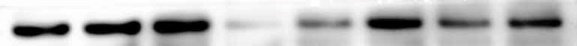


GAPDH


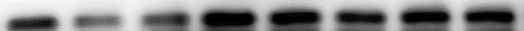


**Figure 5.u**

**Tumor specimen (target at all lane)**

REST

**
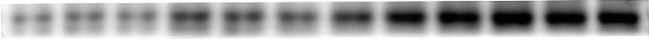
**

GAPDH

**
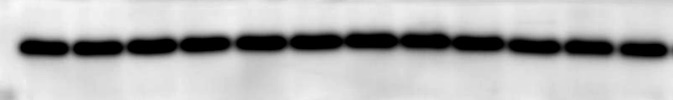
**

**Figure 6.a**

**U87 and U87R (target at lane 7,8,9,10)**

REST

**
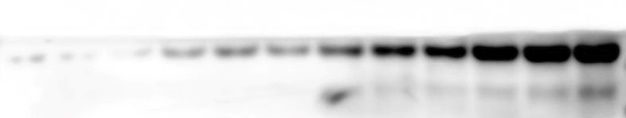
**

GAPDH

**
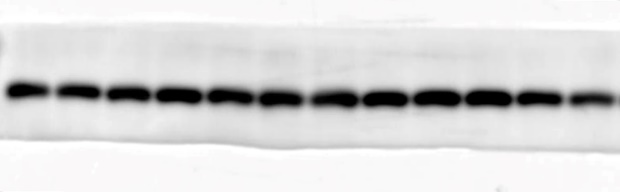
**

**Figure 6.b**

**GSCm01 and GSCm01R (target at lane 5,6,7,8)**

REST

**
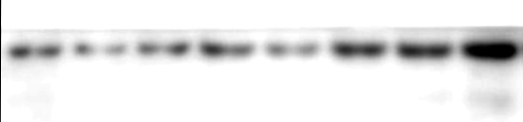
**

GAPDH

**
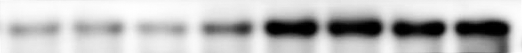
**

**Figure 6.r**

**GSCm01R (target at lane 5,6,7,8)**

CD133

**
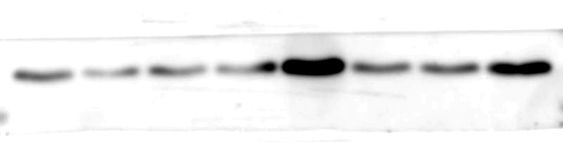
**

SOX2

**
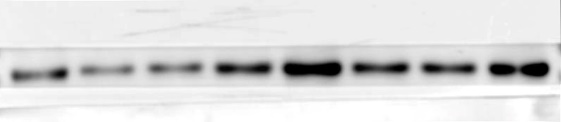
**

OCT4

**
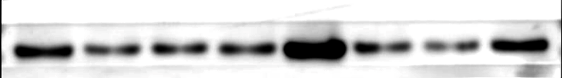
**

Nanog

**
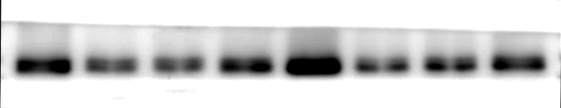
**

Nestin

**
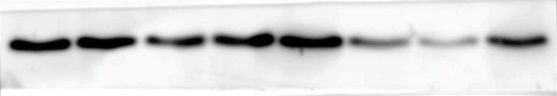
**

GAPDH

**
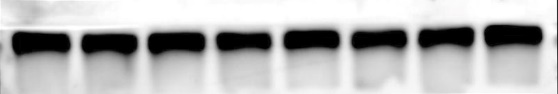
**

**Figure 7.f**

**U87R (target at lane 2,3,4,5,6)**

SLC40A1

**
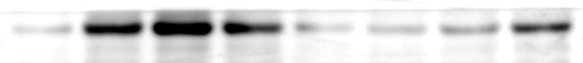
**

GAPDH

**
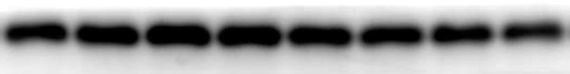
**

**GSCm01R (target at lane 4,5,6,7,8)**

SLC40A1

**
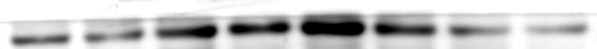
**

GAPDH

**
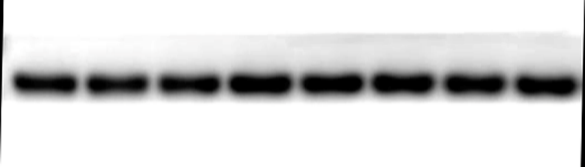
**

**Figure7.i**

**U87R (target at lane 7,8,9)**

(IP SLC40A1) LRSAM1

**
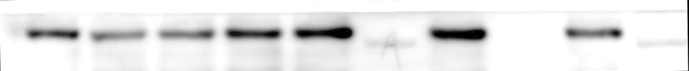
**

(IP SLC40A1) SLC40A1

**
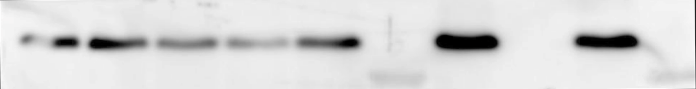
**

**GSCm01R (target at lane 7,8,9)**

(IP LRSAM1) SLC40A1

**
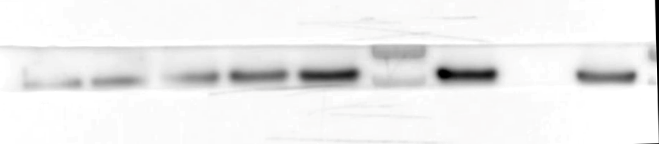
**

(IP LRSAM1) LRSAM1

**
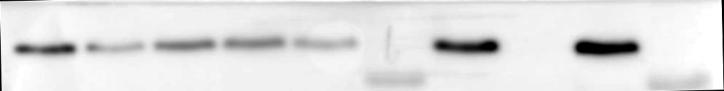
**

**Figure 7.j**

**U87R (target at lane 3,4,5,6,7)**

SLC40A1

**
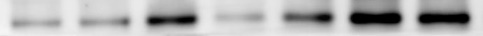
**

GAPDH

**
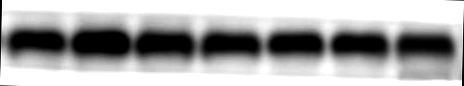
**

**GSCm01R (target at lane 3,4,5,6,7)**

SLC40A1

**
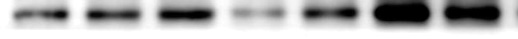
**

GAPDH

**
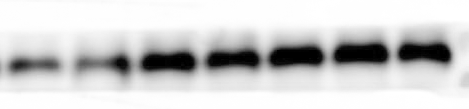
**

**Figure 7.l**

**U87R (target at all lane)**

IB

**
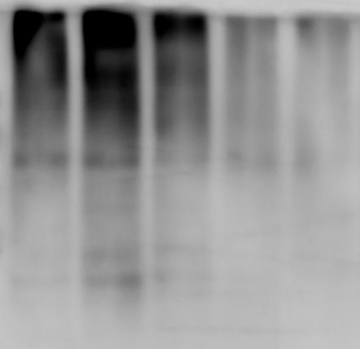
**

Flag

**
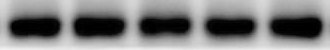
**

**GSCm01R (target at all lane)**

IB

**
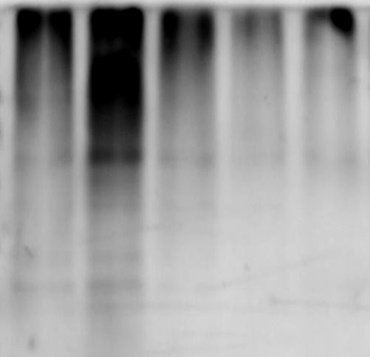
**

Flag

**
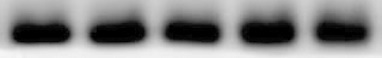
**

**Figure 7.m**

**U87R (target at all lane)**

SLC40A1

**
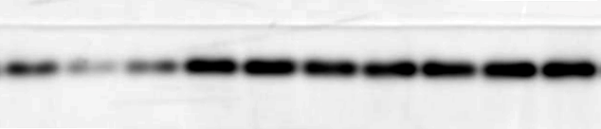
**

GAPDH

**
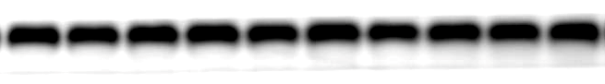
**

**GSCm01R (target at all lane)**

SLC40A1

**
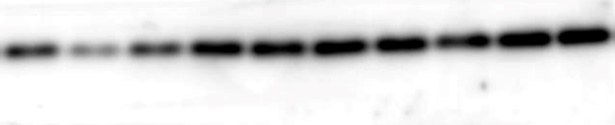
**

GAPDH

**
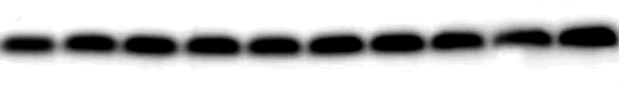
**

**Figure 7.n**

**GSCm01R (target at all lane)**

LRSAM-EV


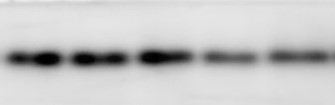


LRSAM-OE


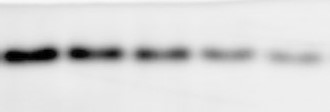


LRSAM-NC


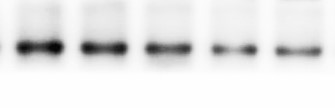


LRSAM1-KD1


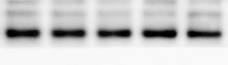


LRSAM1-KD2


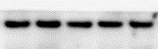


GAPDH

**
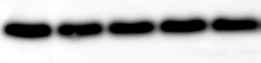
**

**Figure 7. o**

**U87R (target at all lane)**

LRSAM-EV


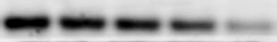


LRSAM-OE


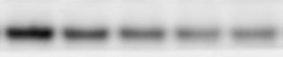


LRSAM-NC


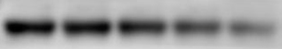


LRSAM1-KD1


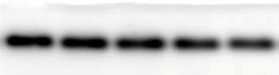


LRSAM1-KD2


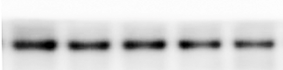


GAPDH

**
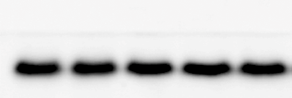
**

**Figure 7. q**

**U87R (target at lane 1,2,3,4)**

SLC40A1


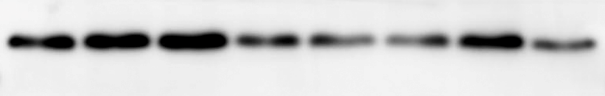


GAPDH

**
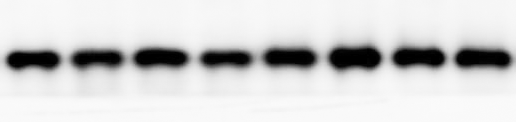
**

**Figure 7. r**

**GSCm01R (target at lane 1,2,3,4)**

SLC40A1


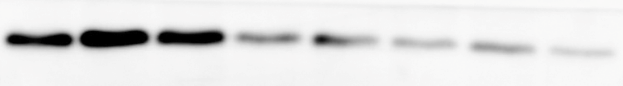


GAPDH

**
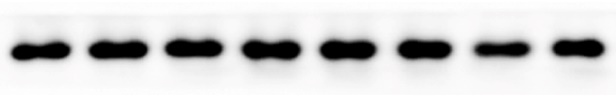
**

**Figure 7. s**

**U87R (target at lane 1,2,3,4)**

SLC40A1


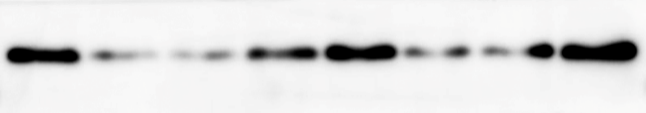


GAPDH

**
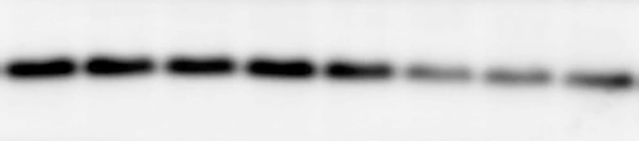
**

**GSCm01R (target at lane 1,2,3,4)**

SLC40A1


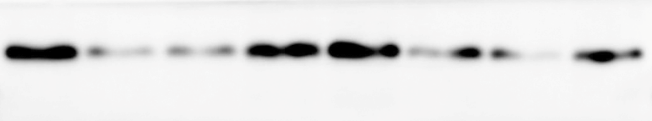


GAPDH

**
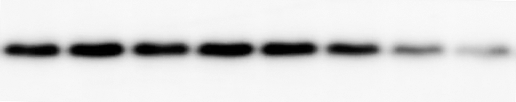
**

**Figure 7. t**

**U87R (target at lane 4,5,6,7)**

SLC40A1


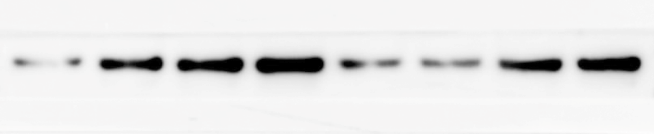


GAPDH

**
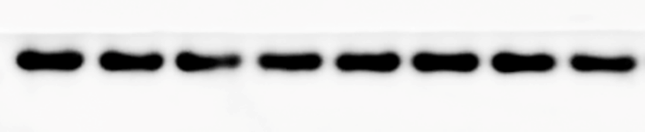
**

**GSCm01R (target at lane 1,2,3,4)**

SLC40A1


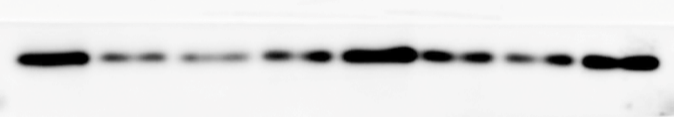


GAPDH

**
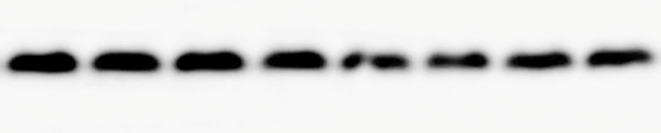
**

**Supplementary Figure 3.h**

**U87R (target at lane 1,2,3,4,5)**

LRSAM1

GAPDH

**Supplementary Figure 3.i**

**GSCm01R (target at lane 1,2,3,4,5)**

LRSAM1

GAPDH

**Supplementary Figure 4.d**

**U87R (target at lane 1,2,3,4)**

LRSAM1

GAPDH

**GSCm01R (target at lane 4,5,6,7)**

LRSAM1

GAPDH

**Supplementary Figure 5.n**

**GSCm01R (target at lane 1,2,3,4)**

CD133

SOX2

OCT4

Nanog

Nestin

GAPDH

**Supplementary Figure 6.a**

**U87R (target at all lane)**

IB

Flag

**Supplementary Figure 6.b**

**GSCm01R (target at all lane)**

IB

Flag

**Supplementary Figure 6.c**

**U87R (target at all lane)**

SLC40A1

GAPDH

**Supplementary Figure 6.d**

**GSCm01R (target at all lane)**

SLC40A1

GAPDH

**Supplementary Figure 6.e**

**U87R (target at all lane)**

SLC40A1(DMSO)

SLC40A1(Erianin)

GAPDH

**Supplementary Figure 6.f**

**GSCm01R (target at all lane)**

SLC40A1(DMSO)

SLC40A1(Erianin)

GAPDH
